# Exposure to benzene, toluene, ethylbenzene, and xylene (BTEX) at Nigeria's petrol stations: a review of current status, challenges and future directions

**DOI:** 10.3389/fpubh.2024.1295758

**Published:** 2024-03-25

**Authors:** Emmanuel Ademola Anigilaje, Zaheer Ahmad Nasir, Christopher Walton

**Affiliations:** School of Water, Energy and Environment, Cranfield University, Cranfield, United Kingdom

**Keywords:** narrative review, BTEX exposure, cancer and non-cancer risks, petrol stations, Sustainable Development Goals, Nigeria

## Abstract

**Introduction:**

In Nigeria, because of increasing population, urbanization, industrialization, and auto-mobilization, petrol is the most everyday non-edible commodity, and it is the leading petroleum product traded at the proliferating Nigeria's petrol stations (NPSs). However, because of inadequate occupational health and safety (OHS) regulatory measures, working at NPSs exposes petrol station workers (PSWs) to a large amount of hazardous benzene, toluene, ethylbenzene, and xylene (BTEX) compounds.

**Methods:**

Studies on BTEX exposures among Nigerian PSWs are scarce. Thus, constraints in quantifying the health risks of BTEX limit stakeholders' ability to design practical risk assessment and risk control strategies. This paper reviews studies on the OHS of Nigerian PSWs at the NPSs.

**Results:**

Although knowledge, attitude, and practices on OHS in NPSs vary from one Nigeria's study setting to another, generally, safety practices, awareness about hazards and personal protective equipment (PPE), and the use of PPE among PSWs fell below expectations. Additionally, air quality at NPSs was poor, with a high content of BTEX and levels of carbon monoxide, hydrogen sulfide, particulate matter, and formaldehyde higher than the World Health Organization guideline limits.

**Discussion:**

Currently, regulatory bodies' effectiveness and accountability in safeguarding OHS at NPSs leave much to be desired. Understanding the OHS of NPSs would inform future initiatives, policies, and regulations that would promote the health and safety of workers at NPSs. However, further studies need to be conducted to describe the vulnerability of PSWs and other Nigerians who are occupationally exposed to BTEX pollution. More importantly, controlling air pollution from hazardous air pollutants like BTEX is an essential component of OHS and integral to attaining the Sustainable Development Goals (SDG) 3, 7, and 11.

## 1 Background and purpose

The petroleum products traded at Nigeria's petrol stations (NPSs) are engine lubrication oil, petrol, diesel, kerosene and cooking gas, but petrol is the leading commodity (1). In 2018, there were 29,197 petrol stations in Nigeria (2). This proliferation is attributed to the country's increasing population, urbanization, industrialization, auto-mobilization, and energy use (3, 4). The daily petrol consumption in Nigeria is about 93 million liters (5). With 198 million people and a motor vehicle population of 11,760,871 in 2018, Nigeria has 0.06 vehicles per person (6). However, most (97.4%) of the available vehicles in Nigeria are imported second-hand vehicles (7), which have been associated with low energy efficiency, high fuel consumption, and high emission of greenhouse gases (GHGs), including carbon dioxide, carbon monoxide, nitrogen oxides, unburned hydrocarbons, and particulates such as soot and ash (8–11). Furthermore, within Nigeria's context of perennial inability to generate, transmit and distribute sufficient electricity (12, 13) and the unaffordability of zero-emission electric vehicles (ZEEVs) (14), Nigerians will continue to depend on gasoline and diesel for their auto-mobiles, and for fuelling their electric generators at homes and businesses (4, 15–17). In Nigeria, petrol station workers (PSWs) typically dispense fuel, unlike self-service dispensers, which are more common in advanced countries (4). Thus, NPSs are an indispensable sector of Nigeria's economic activities, where humans and petroleum products will continue to interact. Unfortunately, the petrol being officially sold in Nigeria has a permissible content of benzene of 2% v/v^1^ compared to 1% (v/v) in Europe (18) and 0.62% (v/v) in the United States (19). In general, petrol contains about 2–18% of benzene, toluene, ethylbenzene and xylene (BTEX) (20, 21). BTEX harm the environment and human health because of their properties and residence times in the atmosphere (22). Nevertheless, BTEX must be added to unleaded gasoline and diesel to act as an antiknock and lubricating agent to improve machine efficiency (23, 24).

BTEX is a mono-aromatic mixture found in natural and anthropogenic sources (25). The natural sources of BTEX are natural gas and petroleum deposits, volcanoes, and wildfires (25). The anthropogenic sources include emissions from aircraft and cigarette smoke; however, in urban areas, the combustion of gasoline and diesel fuels, especially for motor vehicles, constitutes an essential source of BTEX (25–27). Additional sources of BTEX in urban air are emissions from gas stations and small-scale industries that use chemical compounds containing BTEX (paint, adhesives, etc.) (28, 29). BTEX is also a common additive to some chemical intermediates, pharmaceutical products, and consumer products (inks, cosmetics) (30).

BTEX is the main representative of volatile organic compounds (VOCs) (31). By definition, VOCs are photochemically reactive species with high vapor pressure in the Earth's atmosphere (32).VOCs are hazardous air pollutants (HAPs) because they are harmful to the environment and human health due to their properties and residence times in the atmosphere, which can last from a few minutes to several months (22, 23, 33). The residence time of BTEX in the atmospheric air depends on air dispersion and photochemical decomposition with hydroxyl (.OH) oxidant and chloride radicals (34). Thus, apart from diffusion and distance from the source (31, 35), BTEX concentration in the atmospheric air also depends on the BTEX content of the fuel (36) and on atmospheric hydroxylation, which is dependent on temperature (37), seasonal, geographical, altitudinal and diurnal variations (38). According to Atkinson et al. (39), benzene has an estimated lifetime reaction with OH radical of 9.4 days, followed by toluene (1.9 days), ethylbenzene (1.6 days), o-xylene (0.8 days) and m, p-xylene (0.6 days). In other words, while xylene is considered a highly reactive species, ethylbenzene and toluene are less reactive, and benzene is a relatively stable species (40). The concentration of BTEX is usually lower in warmer months due to the strong photolysis and the dilution caused by the increase in the depth of the mixing layer (41). Furthermore, the concentration of BTEX tends to increase during winter due to the frequent occurrence of the inverse temperature layer (31). BTEX accumulation in the air is also higher during cloudy days than sunny days due to lower temperatures and light intensity (31). The BTEX contents are also affected by factors such as prevailing wind direction and wind velocity (42).

Although exposure to BTEX is usually a simultaneous exposure to all its constituent parts, the harmful impacts on human health are better appreciated by considering the individual impacts of each constituent, as multiple human epidemiological studies are available (25). Table 1 summarizes the acute and chronic effects of BTEX in humans (43–46). TEX also forms secondary air pollutants, including ozone, ultra-fine particulate matter, and polycyclic aromatic hydrocarbons that contribute to ill health in humans (31, 47–50).

**Table 1:** The acute and chronic effects of BTEX in humans.

**Chemical**	**Routes of exposure**	**Acute**	**Chronic**	**IARC Carcinogenicity Class**
Benzene	Inhalation, ingestion, skin, and eye contact. Exposure is mainly by inhalation.	Symptoms include drowsiness, dizziness, headaches, skin irritation, respiratory tract, and, at high levels, unconsciousness.	Reduced red blood cells and aplastic anemia in occupational settings. High levels of exposure to inhalation can affect reproductive function in women	Carcinogenic to humans, Group 1: Acute myeloid leukemia and acute non-lymphocytic leukemia are caused by it. All routes of exposure are carcinogenic
Toluene	Despite its potential for skin absorption, toluene is primarily absorbed through inhalation and ingestion.	It mainly affects the central nervous system (CNS) in humans. CNS dysfunction and narcosis, including fatigue, sleepiness, headaches, and nausea	High level exposures cause CNS depression. Others are irritation of the upper respiratory tract and eyes, sore throat, dizziness, and headache. Newborns of pregnant women exposed to high inhalation levels can have problems with attention and mild abnormalities of the head, face, and limbs.	Group 3: Not classifiable as to its carcinogenicity to human
Ethylbenzene	Inhalation, ingestion, skin, and eye contact. Exposure is mainly by inhalation.	Low acute toxicity to humans. Irritation of the eyes and throat, chest tightness, dizziness, and vertigo.	An increase in the mean number of lymphocytes and a decrease in hemoglobin levels	Group 2B: Possibly carcinogenic to humans
Xylene	Inhalation, ingestion, skin, and eye contact. Exposure is mainly by inhalation	Irritation of the eyes, nose, and throat, vomiting and diarrhea, and neurological effects	CNS symptoms include headache, dizziness, fatigue, tremors, and in-coordination. Also affect the respiratory, cardiovascular, and renal systems.	Group 3: Not classifiable as to its carcinogenicity to humans

IARC, International Agency for Research on Cancer.

Human occupational exposure to BTEX from petrol is a significant health concern, and pieces of evidence abound to prove that PSWs are more at risk of BTEX exposure and health hazards (21, 51, 52). Quantifying BTEX exposures and the health risks among PSWs have been of research interest in Asia, Europe, Canada, and North America (18, 52–64). The results of these studies indicated that PSWs were at a higher risk of adverse cancer and non-cancer health risks (52, 56, 60, 62, 64–66). Although the petroleum oil and gas industry contributes about 9% to Nigeria's gross domestic product (GDP) (67), assessing BTEX exposure among PSWs is a rare research focus in Nigeria. This dearth of data exists despite prevailing conditions that expose PSWs in Nigeria to a high volume of BTEX. For example, the dispensing pumps at Nigeria's filling stations are often powered by gasoline-electric generators, which add to the ambient air pollution at the filling stations. In addition, most of these dispensing pumps have no mechanism for vapor recovery (68, 69), and some of Nigeria's PSWs work for long hours daily ranging from 10 h (69) to more than 12 h (70). Exposure to petrol/BTEX is expected through the skin via inadvertent spills on the body while dispensing petroleum products as the use of personal protective equipment (PPE) is uncommon (16, 71–73). Exposure to BTEX through the gastrointestinal tract also happens due to poor personal hygiene as PSWs on working shift take their meals without washing their hands (16, 69), and PSWs occasionally siphon fuels from the tanks of automobiles (70, 74).

Furthermore, although the four Nigerian crude oil refineries (Port Harcourt I and II, Warri, and Kaduna) run by the state-owned Nigerian National Petroleum Corporation (NNPC) can process 445,000 barrels of crude oil daily, they operate for < 50% of their capacity for years before they were shut down in 2020, having reached zero refining activity in 2019 (75). As of 2017, the total demand for petroleum products in Nigeria was 750,000 barrels per day, already more than the refineries' 445,000 barrels capacity (76). Consequently, 70–80% of the country's petroleum products are imported to meet the national demands (76). Reasons for the underutilization of the national refineries include poor governance, lack of major turnaround maintenance, vandalization of pipelines supplying crude oil to refineries and pipelines carrying petroleum products from the refineries, and inappropriate regulations of the price of the petroleum products that lead to under-recovery of crude oil cost (76). West Africa imports around 50% of its fuels from Amsterdam, Rotterdam, and Antwerp (“ARA” region) (77). However, 80% of the diesel exported from ARA to Africa has sulfur content at least 100 times above the European standard (77). Africa's weak fuel standards enable European traders to use low-quality, cheap blend-stocks to produce low-quality “African Quality” fuel, which damages health with high sulfur, aromatics, and benzene levels (77, 78). Thus, while countries in West Africa export crude oil with low sulfur content, they import petroleum products with high sulfur content from Europe and the US (77). Since the report of Gueinat et al. (77), five West African countries (Nigeria, Ghana, Benin, Togo, and Cote d'Ivoire) announced that they will ban the import of high-sulfur fuel (79). However, Nigeria's current specification for sulfur content in petrol remains at 150 part per million (ppm)^2^, value that is still higher than the European limit of 10 ppm (77).

Nigeria's leading petrol and diesel imports are from the Netherlands, Belgium, India, Norway, and the United Kingdom (80). However, imported petrol from Antwerp in February 2022 was found to have an excess amount of methanol, causing engine damage in vehicles in Nigeria (80). This highlights poor quality checks for petrol specification at load ports and in Nigeria and the need for stringent regulations to ensure the safety and quality of imported products. Furthermore, before 2004, Nigeria was one of the countries with high Tetraethyl lead (TEL) concentration as an octane promoter in its gasoline. However, Nigeria adopted the “Phasing-out leaded gasoline in Nigeria's Initiative” of the World Bank Clean Air Initiative. Nigeria adopted a two-step approach, reducing to 0.15 g Pb/l from 0.2 g Pb/l by the end of 2002 and a total phase-out of leaded gasoline by 2004 (81, 82). Although Nigeria was officially acknowledged to have phased out leaded gasoline in 2004, some Nigerian investigators (83–89) have documented higher blood levels of lead among Nigerian PSWs compared to controls that were not occupationally exposed to gasoline. These findings may not be unexpected as gasoline in Nigeria still contains a lead specification of 50 ppm (see text footnote 2).

To inform future initiatives, policies, and regulations that will safeguard Nigeria's PSWs, a better understanding of the occupational health and safety (OHS) of NPSs is required. Specifically, the burden and the challenges of exposure to BTEX at NPSs must be well-documented. Unfortunately, the inability to quantify the health risks (cancer and non-cancer risks) of BTEX will continue to limit Nigerian stakeholders in designing practical risk assessment and risk control strategies. The detection and quantification of air pollution at Nigeria's petrol stations will significantly enhance workplace health and safety standards by pro-actively addressing occupational risk factors that may affect the wellbeing of employees. It will enable swift and resolute measures at every level, including petrol stations, regulatory bodies, and policy-makers, to avert work-related illnesses and injuries and foster optimal workers' health. Studies on OHS at NPSs are essential for stakeholders to establish a baseline, track progress, draw comparisons, and advocate for risk control strategies.

While prefacing a background of Nigeria's geography and energy mix and Nigeria's environmental pollution and climate actions, this paper reviews the existing studies on air quality at NPSs, including observational and analytical studies on OHS of Nigerian PSWs at the NPSs. The review describes the exposure of Nigerian PSWs to BTEX/petrol and other hazards at NPSs. It presents opportunities for a future direction to safeguard the health and safety of Nigeria's PSWs and others who may be occupationally exposed to petroleum products.

### 1.1 Nigeria's geography and population

**Figure 1:**
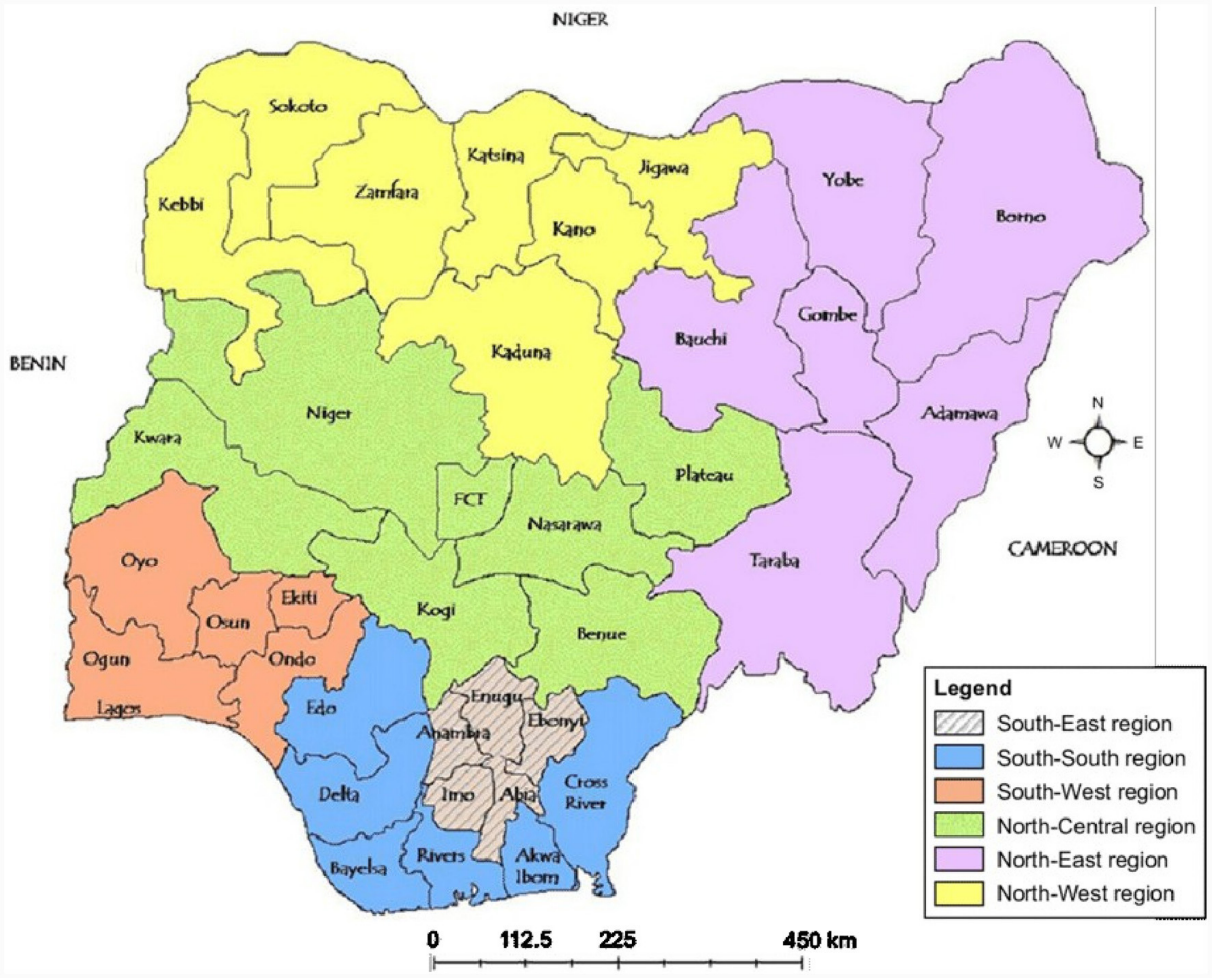
Map of Nigeria showing the 36 states and federal capital territory as well as the six geopolitical zones. Reproduced from Management Commission. niMC enrolment centers. Available from: https://www.nimc.gov.ng/nimc-enrolment-centres/.32. Available via license: Creative Commons Attribution-NonCommercial 4.0 International.

Nigeria has an area of 923,768 square kilometers and is the most populous country in Africa, with an estimated population of about 225 million people in 2022^3^. Nigeria comprises 36 States and Abuja, the Federal Capital Territory (see text footnote 3). The States are aggregated into six geopolitical zones: North-west (NW), North-east (NE), North-central (NC), South-west (SW), South-east (SE), and South-south (SS) (Figure 1). The countries at Nigeria's borders include the Benin Republic to the West, Cameroon to the East, and the Niger Republic to the North (see text footnote 3). Nigeria is a lower middle-income country (LMIC) (90). As of 2022, Nigeria's GDP is 477 United States Dollars (USD), Gross National Income per capita is 2,140 USD, and the total unemployment rate is 5.8% (90). Nigeria is a major producer and exporter of oil in Africa, with significant crude oil reserves, which stood at 37,448.25 million barrels in 2014 (91). Nigeria's crude oil accounts for about 9% of the total GDP and 96% of its export earnings (67, 92, 93).

### 1.2 Nigeria's energy and electricity mix

Nigeria's primary energy consumption in 2017 was about 1.5 quadrillion British thermal units (94). Natural gas (42%), petroleum, and other liquids (55%) are the major energy consumption, while traditional biomass and waste (wood, charcoal, manure, and crop residues), coal, and renewable energy only accounted for 3% (94). Some Nigerian households use biomass energy to cook in poorly ventilated kitchens (95, 96).

In 2017, Nigeria's generation capacity was 12,664 megawatts (MW), of which 10,522 MW (83%) was from fossil fuels, 2,110 MW (17%) was from hydroelectricity, and 32 MW (< 1%) was from solar, wind, and biomass and waste (94). The solar energy is available to very few Nigerians (97). Net electricity generation was far lower than capacity and was 30.6 billion kilowatt-hours (3,495 MW) in 2017, or about 28% of total capacity (94). Although Nigeria is the continent's largest economy, only 60% of the population had access to electricity in 2018 (94). Most of Nigeria's fossil fuel-derived electricity is from natural gas, and crude oil is mainly used for backup power generation (94). Nigeria does not generate energy from nuclear or geothermal sources (91).

Although Nigeria has ambitious electricity mix targets, generating electricity faces persistent challenges, including inadequate power generation due to financial constraints, and problems with energy transmission and distribution (12, 93, 98, 99). Privatization of generation and distribution has yet to eliminate these problems (12, 98). Transmission challenges include mismanagement issues, poor maintenance, and inefficient grid design (12, 98). Consequently, Nigeria's electricity shortfall is met with diesel and gasoline-powered electric generators at homes and at business centres, with some of these electric generators operating between 15 and 18 h a day (17). A staggering $22 bn (about 5% of GDP) is spent yearly to fuel electric generators in Nigeria (93).

### 1.3 Nigeria's environmental pollution

Because of increasing population and rapid urbanization, Nigeria is replete with many environmental problems, including rapid deforestation, soil degradation and loss of arable land, illegal exploration and refining of crude oil, uncontrolled gas flaring, and ambient and household air pollution [(100); see text footnote 3]. Gas-flaring in Nigeria is seventh in the world (6.6 billion cubic meters of flared gas) producing about 17.76 Mt of CO2 emissions as of 2021 (101). Electric generators are sources of air pollution emitting fine particulate matter (PM) and black carbon from internal combustion of diesel and gasoline. Nigerians also buy fuel in plastic containers for their electric generators from the filling stations (102); air pollution also results from evaporative and spillage losses of diesel and petrol in transit from these plastic containers. Nigeria's efforts at mitigating air pollution from electric generators culminate in the flagging off of the National Generator Emission Control Programme (NGECP) in January 2023 (103). The NGECP involves yearly testing of electric generators for toxic and GHG emissions (103). Other sources of ambient air pollution in Nigeria include the burning of e-waste, emissions from waste incinerators, gaseous emissions from dump sites, and gaseous emissions from industries (104, 105). Thus, many Nigerians suffer pollution-related health problems (106) including cardiovascular diseases, mental health problems, and chronic obstructive pulmonary diseases (pneumonia, emphysema, and bronchitis) (107). Nigeria is the fourth leading country with deaths from air pollution (108). An estimated 114,000 Nigerians die from air pollution each year in Nigeria (108). Air pollution was also reported to be a major risk factor responsible for 15% of under-five mortality in Nigeria (109).

### 1.4 Nigeria's transportation system and transportation-related pollution

The commonest source of BTEX in urban areas is transportation activity resulting from incomplete combustion in motor vehicles (110, 111). The transport sector is Nigeria's greatest carbon-dioxide emitter, accounting for about 60% of total national emissions (94). It comprises road, rail, air, and marine sub-sectors; however, the road transport sector is the primary means of moving goods and people across the country (92, 112). Road transport contributes significantly to the nation's GDP (2.7%) (92). Nigeria's transport system comprises more of the least energy-efficient (road and air transport) system, which emits higher GHGs compared to the most energy-efficient sub-sectors (rail and water transport) (14). Passenger transport dominates road transport, as evidenced by a predominance of privately owned cars and light commercial vehicles (92). In 2018, ownership of road vehicles in Nigeria comprised commercial (57.70%), private (40.98%), and Government and diplomatic (1.32%) vehicles (6, 14). Nevertheless, Nigeria's opportunities for low-carbon transport include the use of biofuels (1.5 billion liters planned capacity), natural gas (187 trillion cubic feet) with the use of Compressed Natural Gas (CNG) fuelled vehicles, electrified transport coal (2.7 billion tons), or natural gas power generation sources (92). Challenges include the facts that Nigeria is yet to develop its biofuels program, a slow transition to expensive energy-efficient vehicles as about 60% of the country's population still lives below the poverty line (92), and slow adoption of ZEEVs as only about 60% of the Nigerian population has access to electricity (94). Moreover, transportation-related air pollution and carbon-dioxide emissions are worsened by the pervasive use of used motor vehicles (cars, trucks, lorries, buses, and motorbikes) in Nigeria (113, 114). In 2023, Nigeria's used car market of 500,000 sales (compared to 13,000 sales for brand-new vehicles), valued at $1.14 billion, constitutes 97.4% of available vehicles in the country (7). This market is estimated to grow by 8.9% in 2024 due to high inflation, declining GDP, and the spike in new car prices (7). Although the Nigeria Customs Service increased the import duty on vehicles from 39.45 to 39.62% to promote domestic manufacturing of vehicles and reduce imported cars, this has not reduced the dependence on used cars (115). Nigeria's import regulation limits used vehicle age to 15 years (116), however, this policy has failed to reduce the number of imported used vehicles (117). The complexities in the regional market for used vehicles in West Africa are such that trade restriction rules in Nigeria are often circumvented by the viable re-exportation of used cars from the alternative import routes from the neighboring countries of Benin and Togo Republics (117, 118). Benin and Togo have no age restriction for used vehicles, and they use a low-import tariff strategy to re-export to Nigeria (117, 118). Launched in January 2023, the National Vehicular Emission Control Programme (NVECP) provides annual testing of vehicles for toxic and GHG emissions (103). Sadly, the use of ZEEVs is uncommon in Nigeria due to high upfront costs, lack of charging infrastructure, lack of technical know-how, and political entrenchment of oil and gas (119). ZEEVs do not produce emissions irrespective of age, compared to internal combustive engine (ICE) vehicles, where emissions intensity can increase over time and more with a lack of maintenance (119). While Nigeria has the potential to import used ZEEVs, policymakers worry about the impact on Nigeria's crude oil exports (120). Nonetheless, deploying ZEEVs could lower emissions and remove the burden of petroleum subsidies Nigeria has to pay (120).

### 1.5 Nigeria's climate action

Nigeria is a party to the United Nations Framework Convention on Climate Change, which aims to limit the Earth's warming to 1.5 degrees Celsius (2.7 Fahrenheit). Nigeria commits to reducing carbon emissions by 20% by 2030 (121) and achieving net-zero carbon emissions by 2060 (122). Nigeria's commitment to global climate mandates includes policies like the Nationally Determined Contribution, National Climate Change Policy, National Climate Change Council, and Energy Transition Plan (123). However, Nigeria needs $1.9 trillion to achieve net-zero emissions in 2060, relying on international climate finance (123). The Conference of Parties (COP28) in Dubai agreed to four pillars: fast-tracking a just, orderly, and equitable energy transition from fossil fuel, fixing climate finance, focusing on people, lives and livelihoods, and underpinning everything with total inclusivity (124). At COP28, Nigeria secured over $400 million in commitment to the loss and damage fund and signed several commitments to establish solar panel and lithium battery manufacturing factories in Nigeria (125, 126). A lingering challenge remains that Nigeria is a fossil fuel-dependent developing country (FFDC) which rely on fossil fuel income and carbon intensive industries like the transport system. Nigeria's ability to successful transition will therefore depend on its capacity to diversify its assets and revenues (127). Nevertheless, Nigeria is already on the right tract for a just energy transition as the country has removed subsidies for fossil fuel consumption in 2023 (128).

## 2 Materials and methods

A comprehensive search of articles, abstracts and proceedings of conferences published in English in peer-reviewed academic journals and conferences was made. Electronic databases, including Medline, Embase, Scopus, Google Scholar, and African Journal Online (AJOL) were searched with no date restriction for ALL related works on the research interest. The keywords used for searches were “exposure to BTEX among petrol station attendants in Nigeria”; “exposure to volatile organic compounds at petrol stations in Nigeria”; “health hazards of petrol station attendants in Nigeria”; “knowledge of safety practices in filling stations in Nigeria”; “knowledge, attitude and practices on occupational health and safety in Nigeria's petrol stations”; “health effects of petrol fumes in Nigerian filling station attendants”; “petrol station attendants in Nigeria”; “symptoms among petrol station attendants in Nigeria”; “hazards among fuel station attendants in Nigeria” and “assessment of occupational safety and health in petrol stations in Nigeria”. When related articles were identified, the references cited by these articles were also searched for relevant articles.

**Figure 2:**
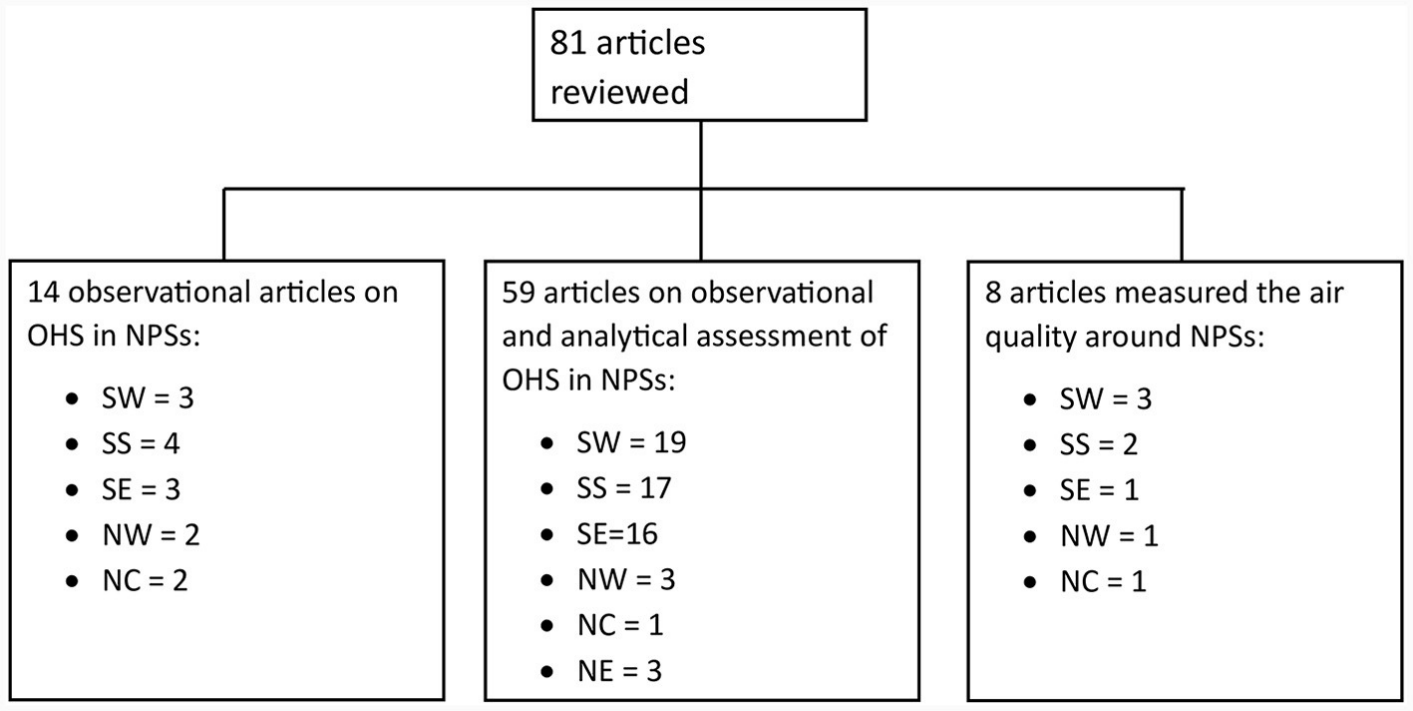
A diagrammatic sketch of the reviewed 81 articles. SW, southwest; SS, southsouth; SE, southeast; NW, northwest; NC, northcentral; NE, northeast; OHS, occupational health and safety; NPS, Nigeria's petrol stations.

Figure 2 depicts the diagrammatic sketch of the 81 reviewed articles. The first group was observational research that included studies of knowledge, attitude, and practices (KAPs) of occupational health and safety (OHS) in NPSs, for which questionnaires and checklists were used. The second was observational and analytical studies for which blood samples were taken for petrol fumes impacts on organ-systems of the Nigerian PSWs (haematologic, immunologic, hepatic, renal), or for which ultrasound scans were done, or physiologic (respiratory function indices) impacts were measured among the Nigerian PSWs. The third was those studies that measured ambient air quality in and around the NPSs.

## 3 Results

### 3.1 Observational studies

Table 2 summarizes the findings of the observational studies.

**Table 2:** Summary of the observational studies.

**References**	**Study Site**	**Subjects and controls, number of petrol stations sampled**	**Major findings**
Afolabi et al. (129)	Ile-Ife, Osun State, Southwest	Subjects: 54 FSAs; No controls. 7 PSs.	94% of subjects were aware of safety measures. Fire extinguisher was the commonest safety measure (54%). Fire (94.4%) and armed robbery (27.8%) were the commonest hazards. Exposure to petrol fumes was reported in 7.4%. Setbacks from the road and residential areas did not conform with the regulations of the DPR in 90 and 48% of the filling stations, respectively
Aguwa et al. (70)	Abia, Abia State, Southeast	Subjects: 170 filling station pump operators; no controls. 170 PSs.	85.3% were aware of occupational hazards. Common hazards and health symptoms included fumes inhalation in 38 (22.4%), accidents in 37.1%, itchy eyes in 48.2%, headaches in 22.4% and vomiting in 14.1%. 89.4 and 12.4% used overall and gloves as PPE, respectively. Safety measures were signage of “no smoking” (94.1%), “switch off cell phone” (92.9%) and “turn off engine” (7.6%). Safety policies existed in 70% of PSs. Knowledge of hazards, number of years at work and punishment for non-compliance to safety rules were associated with regular use of PPEs (*P* = 0.01). Gender and level of education were not associated with the use of PPE
Ahmed et al. (15)	Minna, Niger State, Northcentral	Subjects: 50 PSAs; No controls. 50 PSs.	Fuel spillage (90%) and health hazards (54%) were common. Ninety percentage of subjects knew safety measures, but only 35% received formal safety training. The fire extinguisher was the commonest (90%) safety measure. Most (90%) of the PSs did not conform to the DPR rules on setbacks from the road and residential areas (< 30 meters).
Akodu et al. (130)	Lagos, Lagos State, Southwest	Subjects: 285 FSAs; no controls. PS number not stated.	LBP over 12 months was reported in 84.60%. The LBP intensity was moderate (71.23%), mild (17.54%) and severe (11.23%) on a verbal rating scale. There was a relationship between age (*p* = 0.0001), gender (*p* = 0.0001) and years of experience (*p* = 0.0001) of FSAs and LBP. Significantly more males (230, 80.7%) experienced LBP. A majority (171, 60%) of the respondent reported that prolonged standing was the activity that predisposed them to LBP
Kakwi (131)	Kaduna South, Kaduna State, Northwest	Subjects: 212 PPAs; no controls. 27 PSs.	Awareness of fire extinguishers as a safety measure was 99.1 and 92% for PPE, but usage was lower, at 76.9% for fire extinguishers and 77.8% for PPE. The level of education, work experience and attitude, were the best predictors for compliance with safety practices
Okafoagu et al. (16)	Sokoto, Sokoto State, Northwest	Subjects: 108 petrol pump attendants; no controls. 40 PSs.	Sixty-two (59.0%) had knowledge of hazards; 72(72.4%) were aware that VOCs were harmful to health, 92.4% knew that no smoking should take place at filling stations, and 83% were aware of the need to turn off vehicle engine while refueling. 2.8% used hand gloves, and 19.4% used an apron always. The awareness of the use of PPE was 75%, but only 34.3% used a form of PPE
Johnson and Umoren (69)	Uyo, Akwa Ibom State, Southsouth	Subjects: 215 PSAs; no controls. 81 PSs.	Common hazards acknowledged were petrol fumes inhalation 145 (67.4%) and confrontation from customers 112 (52.1%). The commonest health problems were headache (53.6%) and LBP (33.3%). A significant relationship was found between headache, nausea, cough, and inhalation of petrol vapor (*p* < 0.01) or car exhaust fumes (*p* < 0.05). Awareness about PPE was 30.7%, while use was 7.0%. Hand washing after contact with fuel was practiced by 73.5% of the Subjects. Only 4.2% reported ever undergoing pre- or post-employment medical examination
Moke (73)	Abraka, Delta State, Southsouth	Subjects:35 PSAs; no controls. 5 PSs.	Subjects worked for at least 2 years; some (18–51.4%) worked for more than 8 h daily, most (51.4%) used PPE during working hours, and few (13–37.1%) used PPE regularly. Health problems were cough 5 (14.3%) & breathing difficulty 4 (11.4%).
Chijoke (132)	Enugu, Enugu State, Southeast	Subjects: 400 PSAs; no controls. PS number not stated.	Hazards reported were inhalation of petrol fumes, customer confrontation, armed robbery, and noise. Health symptoms were headache, low back pain, sciatic pain, muscle spasm, eye irritation, dizziness, cough, and nausea. Awareness about the correct use of PPE was inferior prior to the intervention but increased up to 94.5% immediately after the intervention
Emokpae et al. (71)	Benin, Edo State, Southsouth	Subjects: 90 FPAs; No Controls. 90 PSs.	The majority (95.6%) were aware of at least one type of PPE, 24.4% were aware of the health hazards of petroleum products, and a few (18.8%) used a form of PPE. The commonest health hazards were inhalation of petrol fumes (80%) and skin contamination of petroleum products (20%). The awareness of health hazards and use of PPE correlated positively with educational status and duration of employment, respectively
Emeka and Achalu (133)	Port-Harcourt, Rivers State, Southsouth	Subjects: 767 managers and employees; no controls. 270 PSs.	The study showed no significant difference in exposure to fire safety hazards based on gender. There was a significant difference in adherence to fire safety measures based on age (*p* < 0.05), educational status (*p* < 0.05) and marital status (*p* < 0.05).
Ogunkoya et al. (4)	Sagamu, Ogun State, Southwest	Subjects: 106; no controls. PS Not stated.	The majority, 67 (63.2%) of PPAs, strongly agreed or agreed that they were trained and retrained on safety measures by their employer. 50 (47.2%) PPAs agreed or strongly agreed that employers enforced safety policies. 7 (25.4%) PPAs strongly agreed or agreed that employers provided PPE. 23 (21.7%) used hand gloves, 37 (34.9%) used boots, 17 (16%) used face masks and only 02 (1.9%) used face shields or goggles at work (less than half of PPAs used one form of PPE or another)
Lawal (134)	Ilorin, Kwara State, Northcentral	Subjects: 96 RPS employees; Controls 200 individuals within Kwara State University. 36 PSs.	Poor OHS management practices observed at the selected retail petrol stations and poor awareness and knowledge of health risks related to RPS among RPS owners. The public and environmental health officers were aware of the health and environmental risks associated with RPSs. No statistically significant difference between the retail petrol station employees and the general population's quality of life noted on the SF-36 questionnaire.
Kassy et al. (135)	Enugu, Enugu State, Southeast	Subjects: 210 PPAs aged 17–35 years and a mean age of 23.55 ±5.43; no controls. 105 PSs.	Most (75%) had good knowledge, while 64.3% had poor risk perception of occupational hazards. The commonest hazards were fuel inhalation (81.0%) and fuel splashes (81.4%). 46.7% of subjects used PPE. Most PSs had functional fire extinguishers (99.0%) and sand buckets (98.1%), while 36.2% had muster points. 40 and 76.2% of PSs had inadequate residential and road setbacks, respectively

OHS, occupational health and safety; RPS, retail petrol station; FSA, filling station attendant; PSA, petrol station attendants; PS, petrol station; PSs, petrol stations; DPR, department of petroleum resources; PPEs, personal protective equipment; PPAs, petrol pump attendants; LBP, low back pain; VOCs, volatile organic compounds; AOR, adjusted odd ratio; CI, confidence interval; OR, odds ratio; AOR, adjusted odds ratio; CI, confidence interval.

Awareness of safety measures among PSWs varied from 63.2% in Sagamu (4) to 99.1% in Kaduna (131). The commonest safety measures in operations were signage of no-smoking in 92.4% (16) and fire extinguishers in 99% (135). Hand-washing after contact with petrol was also prominent in 73.5% of PSWs in Uyo (69).

Awareness of hazards among PSWs at the NPSs ranged from 24.4% in Benin (71) to 85.3% in Aba (70). Petrol fumes inhalation as a hazard among PSWs was recognized by 81% of the PSWs in Enugu (135), and by 7.4% of PSWs in Ile-Ife (129). Other hazards recognized were skin contamination by petrol (20%) (71), accidents at the petrol station (37.1%) (70), armed robbery (27.8%) (129), fuel spillage (90%) (15), and fire (94.4%) (129). In the Okafoagu et al. (16) study, PSWs recognize petrol fumes as a health hazard containing volatile organic compounds.

Of the health symptoms experienced by the PSWs, the common ones were headaches (53.6%) and low back pain (33.3%) (69), itchy eyes (48.2%) and headaches (22.4%) (129), and cough and difficulty in breathing (73). The work of Akodu et al. (130) was solely on the burden of low back pain (LBP) among PSWs in Lagos, and the majority (84.6%) experienced LBP over 12 months of working at the petrol stations. The majority (60%) of the respondents also acknowledged that prolonged standing was the activity that predisposed them to LBP (130).

Concerning awareness of personal protective equipment (PPE) among PSWs at NPSs, it was 25.4 and 30.7% in Sagamu (4) and in Uyo (69), respectively. Awareness about PPE availability was 75, 92, and 95.6% in Sokoto (16), Kaduna (131), and Benin (71), respectively. The use of PPE ranged from 7% in Uyo (69) to 89.4% in Aba (70). The common forms of PPE used were overalls (89.4%) (70) and boots (46.7%) (69). Two studies (70, 71) also reported that the regular use of PPE was dependent on workers' awareness of hazards (70), punishment for non-compliance with the use of PPE (70), educational status (71), and the number of years on the job (70, 71). Ahmed and his colleagues (15) in Minna also identified that safety standards are far better in petrol stations owned by major petroleum marketers (conglomerate) compared to petrol stations owned by independent petroleum marketers (IPMs). Lack of adequate staff training, accidents, and fire safety equipment maintenance was noted more commonly at petrol stations owned by IPMs (15).

The only qualitative study by Lawal in Ilorin (134) reported poor OHS management practices observed at the selected retail petrol stations (RPSs) and poor awareness and knowledge of health risks related to RPS among RPS owners. However, the public and environmental health officers know the health and environmental risks associated with RPSs. No statistically significant difference between the RPS employees and the general population's quality of life was noted on the SF-36 questionnaire.

### 3.2 Observational and analytical studies

Table 3 provides a synopsis of the observational and analytical studies. Among PSWs, Nnwanjo and Ojiako (144) in Owerri, Ogunneye et al. (150) in Ijebu-Ode, and Iyanda and Anetor (167) in Ibadan, reported elevated serum alkaline phosphatase (ALP), alanine aminotransferases (ALT) and aspartate aminotransferases (ASP). The serum liver enzymes also increased with years of exposure to petrol vapors (144, 150) and more among the female PSWs (181). Akinosun et al. (141) in Ibadan, on the other hand, reported a reduction of ALP among PSWs in their series.

**Table 3:** Summary of observational and analytical studies.

**Reference**	**Study Site**	**Subjects and controls, number of petrol stations sampled**	**Major findings**
Akintonwa and Oladele (74)	Lagos, Lagos State, Southwest	Subjects: 168 PSAs; Controls 22 students. PS number not stated.	Hemoglobin was 14.5% higher in male Subjects compared to the male Controls; and 0.8% higher in female Subjects compared to the Controls. Serum transaminase levels were higher in subjects compared to controls
Onunkwor et al. (136)^a^	Abeokuta, Ogun State, Southwest	Subjects: 27 PSAs and 21 auto-mechanics; Controls 14 University students. PS number not stated.	The 2-week ascorbic acid supplementation resulted in a significant reduction in BLL in Subjects (57% in male PSAs, 50% in female PSAs). Urinary excretion of Pb increased remarkably in Subjects. Plasma and urine aminolaevulinic acid were reduced significantly by 55 and 57%, respectively. Decreased levels of reduced glutathione (GSH) and Hb observed in Subjects were reversed by ascorbic acid.
Sofola et al. (137)^a^	Lagos, Lagos State, Southwest	Subjects: 26 male depot workers from Apapa (Lagos) and Sagamu (Ogun State) and 23 PSAs from Lagos; Controls were 21 students and staff of the College of Medicine, University of Lagos, Lagos, Nigeria. PS number not stated.	The PEFR values were 386 + 91 and 529 + 94 liters/min in PSAs and Controls, respectively. The value in the Controls was significantly higher than values among PSAs. The value of PEFR in petrol attendants was significantly higher than depot workers. The PEFR was negatively correlated with the duration of employment in PSAs.
Ademuyiwa et al. (138)^a^	Abeokuta, Ogun State, Southwest	Subjects: 99 including 50 auto-mechanics, eight auto-electricians, two battery chargers, two drivers, seven vehicle painters, fifteen panel beaters, seven PSAs, four upholsterers, two spare part/oil sellers and two welders; eleven Controls comprised students and staff of the University. PS number not stated	The risk of cardiovascular disease was higher in the Subjects. Total cholesterol was higher in the Subjects than in the Controls. LDL was higher in the artisans than in the Controls. Blood pressure (systolic and diastolic) and other anthropometric parameters were not significantly different between the Subjects and the Controls.
Ademuyiwa et al. (139)^a^	Abeokuta, Ogun State, Southwest	Subjects: 99 including 50 auto-mechanics, 8 auto-electricians, 2 battery chargers, 2 drivers, 7 vehicle painters, 15 panel beaters, 7 PSAs, 4 upholsterers, 2 spare part/oil sellers and 2 welders; 11 Controls comprised students and staff of the University. PS number not stated.	Administration of a daily dose of 500 mg ascorbic acid for 2 weeks reversed the Pb-induced inhibition of ALAD. Increased EPP levels observed in the Subjects also responded positively to the ascorbic acid supplementation. A significant reduction in BLL was also observed in the Subjects at the end of the 2-week ascorbic acid therapy.
Akintomiwa et al. (140)	Ibadan, Oyo State, Southwest	Subjects: 52 male PSAs; Controls 20 male participants. 20 PSs.	There were no significant differences in the blood levels of RBC, WBC, Hb and PCV between the Subjects and the Controls exposed to petrol by inhalation and oral route. However, PLT was significantly lower by 43% in Subjects compared to Controls. Routes of exposure to petrol did not affect the PLT levels among the Subjects. Petrol exposure did not affect sodium, potassium, creatinine, bilirubin, alkaline phosphate, uric acid, and urea. Elevated SGPT and SGOT levels suggestive of hepatotoxicity were found among the Subjects due to hydrocarbon
Akinosun et al. (141)	Ibadan, Oyo State, Southwest	Subjects: 29 male petrol attendants; 22 Controls sex- and age-matched male staff of the University. Number of PS not stated.	Only ALP was significantly reduced out of all the liver functions (TP, albumin, AST, ALT, ALP, total bilirubin) assessed. Only IgM was significantly elevated out of all the immunoglobulin classes (IgG, IgA, and IgM) determined
Okoro et al. (142)	Calabar, Cross-River State, Southsouth	Subjects: 200 including 100 (50 males, 50 females) T1 Group exposed to petroleum fumes for ≤ 2 years and 100 (50 males, 50 females) T2 Group exposed to petrol fumes for >2 years; Controls 200 (100 males, 100 females) students and shop attendants not occupationally exposed to petrol fumes. Number of PS not stated.	In both genders, RBC (106/mm^3^) was significantly reduced in T1 (4.4 ± 0.13) and T2 (3.85 ±0.07) compared to Controls (4.76 ± 0.01). The WBCs HCT, Hb, and MCHC in both sexes of T1 and T2 were significantly lower than the Controls. The MCH and MCV in the T2 Group were significantly lower than the Controls. The RBC counts, Hb and HCT in the T2 Group were significantly lower than in the T1 Group.
Ademuyiwa et al. (143)^a^	Abeokuta, Ogun State, Southwest	Subjects: 123 including 30 auto-mechanics, 10 auto-electricians, 10 painters, 10 panel beaters, 47 (30 males, 17 females) PSAs, 2 upholsterers, 3 vulcanizers and 11 welders; Controls 25 (15 males, 10 females) staff of the University. Number of PS not stated	AcChE activity was reduced by 39 and 32% in the male and female PSAs, respectively. There was a significant negative correlation between AcChE activity and blood lead levels. Blood pressure and pulse were not significantly different between Controls and Subjects
Nwanjo and Ojiako (144)	Owerri, Imo State, Southeast	Subjects: 20 PSWs; Controls 20 healthy individuals	There was significant increase in ALP, ALT and AST, urea, creatinine, and urinary protein among Subjects compared to Controls
Abama et al. (145)^a^	Abeokuta, Ogun State, Southwest	Subjects: 97 including 25 auto-mechanics, 5 auto-electricians, 8 painters, 10 panel beaters, 35 PSAs (20 males, 15 females), 2 upholsterers, 2 vulcanizers and 10 welders; Controls 25 (15 males, 10 females) staff of the University. Number of PS not stated.	The 2-week ascorbic acid administration resulted in the reversal of lead-induced accumulation of Ca and Mg in the erythrocyte membranes of the Subjects. Ascorbic acid also reversed Pb-induced inhibition of erythrocyte membrane Ca2+-Mg2+-ATPase. Urinary excretion of Ca and Mg was not affected by ascorbic acid.
Udonwa et al. (146)^a^	Calabar, Cross-River State, Southsouth	Subjects: 100 including 50 PSAs and 50 auto-mechanics; Controls 50 persons selected from the general population who did not work in automobile workshops, petrol stations and whose work did not involve petrol. Number of PS not stated.	The mean MetHb value was higher in PSAs (5.8%) than in Controls from the general population (2.7%). PCV was lower in PSAs (30.8%) than in Controls from the general population (40.8%).
Bamgbose et al. (84)	Abeokuta, Ogun State, Southwest	Subjects: 80 PSAs; Controls 35 students of the University of Agriculture, Abeokuta Nigeria.The mean age of the attendants and the students were 32.37 ± 3.40 and 25.57 ± 2.61 years, respectively. Number of PS not stated.	The mean values of BLL were significantly higher at 41.36 ± 2.71 for Subjects and 14.31 ± 2.16 for Controls. The systolic blood pressures were higher among the Subjects compared to Controls, but there was no difference between the diastolic blood pressure.
Alasia et al. (83)^a^	Port Harcourt, Rivers State, Southsouth	Subjects: 190 adults including 42 welders/metal workers, 38 paint/pigment workers, 37 radiator repairers, 37 battery workers and 36 petrol workers; Controls were 80 individuals with limited occupational exposure to lead. Participants were aged 18 to 60 years. Number of PS not stated.	The mean BLL (50.37 ± 24.58 μg/dl) was significantly higher in Subjects than in Controls (41.40 ± 26.85 μg/dl). The mean serum urea (3.06 ± 0.81 mmol/L), creatinine (87.2 ± 14.30 μmol/L) and uric acid (271.93 ± 71.18 μmol/L) in Subjects were significantly higher than the serum urea (2.7 ± 0.84 mmol/L), creatinine (80.68 ± 14.70 μmol/L) and uric acid (231.1 ± 62.70 μmol/L) in the Controls. Creatinine clearance was significantly reduced (98.86 ± 21.26 ml/min/1.72 m^2^) in Subjects compared to the Controls (108.18 ± 25.16 ml/min/1.72 m^2^). BLL correlated positively with blood urea and negatively with serum phosphate.
Onuegbu et al. (89)^a^	Osogbo, Osun State, Southwest	Subjects: 53 (mean age 30.9 + 7.7) including 23 automobile mechanics, 11 battery repair workers and 19 PSAs occupationally exposed to lead-containing products for at least 1 year; Controls 42 male subjects not exposed. Number of PS not stated.	Significantly higher mean plasma concentrations of creatinine, sodium and chloride in Subjects compared with Controls. The mean plasma concentration of urea and BLL were higher in Subjects compared with Controls. No significant difference in the mean values of plasma potassium and bicarbonate levels in Subjects compared with Controls.
Gali et al. (147)	Maiduguri, Bornu State, Northeast	Subjects: 20 petrol hawkers and 35 petrol station attendants; Controls 40 apparently healthy persons	Significantly higher serum levels of AST, ALT, and ALP but lower albumin in petrol hawkers compared to Controls; and higher level of ALP in petrol attendants compared to Controls.
Festus et al. (148)	Owerri, Imo State, Southeast	Subjects: 50 PSAs; Controls 50 “non-petrol station” attendants. Number of PS not stated.	Plasma creatinine and HCO_3_ were significantly higher among the PSAs than the Controls. Plasma Na+ and Cl– were significantly higher in the Controls than in the PSAs. Plasma K+, although higher in the PSAs than the Controls, was not statistically significant.
Ogodo and Ekeleme (149)	Okigwe, Imo State, Southeast	Subjects: 112 petrol station staff and Controls were 112 non-petrol station staff. Number of PS not stated.	Bacterial load in the form of the total aerobic count were 60.56 ± 1.93 × 10^3^ CFU/ml in males and 57.72 ± 2.28 × 10^3^ CFU/ml in females for petrol station staff, while 58.82 ± 2.32 × 10^3^ CFU/ml in males and 59.47 ± 1.93 × 10^3^ CFU/ml in females were obtained for non-petrol station staff. The result revealed no significant difference in bacterial count between petrol station staff and non-petrol station staff.
Ogunneye et al. (150)	Ijebu-Ode, Ogun State, Southwest	Subjects: 30 PSAs; Controls 10 apparently healthy persons not engaged in activities that predispose them to serious contact with petrol fumes. 5 PSs.	Serum AST, ALT, ALP activities and total bilirubin concentration were significantly higher in PSAs with 27–36 months of work experience compared to PSAs with lesser work experience. Serum creatinine, total protein and urea levels were higher among PSAs than the controls.
Adeniyi (151)	Ile-Ife, Osun State, Southwest	Subjects: 99 PPAs; Controls 95 age- and sex-matched security employees of the University who were not occupationally exposed to petrol fumes. Number of PSs not stated.	The pre-bronchodilator per cent predicted FVC and post-bronchodilator per cent predicted FVC were significantly lower in the Exposed compared to the Controls.
Odewabi et al. (152)	Ibadan, Oyo State, Southwest	Subjects: 100 (25 females and 75 males) PSAs; Controls 50 (15 females and 35 males) age, sex and smoking matched who had no known chemical exposure at work. Number of PS not stated.	Exposure to petrol fumes is associated with oxidative stress. Significant elevation of malondialdehyde was associated with a reduction in superoxide dismutase, catalase and glutathione compared to Controls. There was a significant reduction in vitamin E and no significant difference in vitamin C in Subjects compared with Controls. There was a significant decrease in total protein but no significant difference in albumin in PSAs compared with the Controls
Ajugwo et al. (153)^a^	Elele, Rivers State, Southsouth	Subjects: 35 fuel attendants and 35 auto mechanics. Controls: 30 students not occupationally exposed to petrol. The participants aged 18–30 years. Several fuel stations and mechanical workshops.	Fuel attendants exposed to gasoline fumes beyond 2 years have lower PCV, Hb, MCH and MCHC than those exposed for 2 years or less.
Isirima and Angalabiri-Owei (154)	Port Harcourt, Rivers State, Southsouth	Subjects: 100 subjects (40 males and 60 females) aged 18–30. Each gender was further categorized into groups of 10 controls and 30 tests in males and 20 controls, and 40 tests in females. Test Group 1 subjects were exposed to fumes of petroleum products for 2 years and below, while test Group 2 subjects were exposed for more than 2 years.	There was a significant decrease in RBC counts, WBC counts, PCV and Hb concentration in the test Groups 1 and 2 compared to Controls and a significant increase in ALP, ALT, and AST. The odds/odds ratios of Subjects becoming anemic progressively rose from < 1 in the Controls to >1 in test groups.
Ogunkoya (155)	Sagamu, Ogun State, Southwest	Subjects: 106 PPAs; Controls 106 age, sex, weight, and height-matched staff of the University who were not occupationally exposed to petrol fumes. The number of PS was not stated.	The mean values of ventilatory function parameters of PPAs were significantly lower than those of Controls. Among the PPAs, 20 (18.9%) had obstructive defects, and 12 (11.3%) had restrictive defects compared to 14 (13.2%) and 8 (7.5%) in the Control, respectively. The mean ventilatory function parameters of PPAs who smoked (ex and current) were lower than the Controls who smoked (ex and current). The difference was statistically significant for PEFR. The mean ventilatory function parameters in PPAs who never smoked were not significantly lower than in non-smoking controls.
Alli (156)^a^	Gwagwalada, Abuja, Northcentral	Subjects: 64 including 30 auto-mechanics, 13 generator mechanics, 12 fuel attendants and 9 others including battery charger, spray painters; Controls 56 non-occupationally exposed students at the university. Number of PS not stated.	A significantly elevated mean blood level of cadmium (10.46 + 1.05 μg/dl) and lead (48.45 + 7.25 μg/dl) in Subjects compared to cadmium of 2.03 + 0.55 μg/dl and 12.08 + 2.87 μg/dl for lead among the Controls.
Emeji et al. (157)	Port-Harcourt, Rivers State, Southsouth	Subjects: 20 (12 males, 8 females) PPAs; Controls 10 apparently healthy individuals who work within an office setting away from petrol station. 7 PSs.	All the serum electrolytes (in mmol/liter) were higher in Subjects than the Controls including potassium 4.3± 1.8, sodium 151 ± 3.7, chloride 115 ± 14.6, bicarbonate 26 ± 3.3 in the Subjects and potassium 3.5 ± 0.2, sodium 137 ± 2.5, chloride 101 ± 0.3, bicarbonate 21 ± 1.3 in the Controls. All expect potassium were significantly higher in Subjects compared to the Controls.
Uko et al. (158)	Sokoto, Sokoto State, Northwest	Subjects: 100 PSAs with a mean age of 39.48 ± 8.24 years; Controls 50 age and gender-matched non-exposed individuals. Number of PSs not stated.	Hb, PCV, RBC, WBC, MCH and MCHC were significantly lower among Subjects compared to Controls. Subjects who were exposed to petrol for more than 2 years had significantly lower Hb, PCV, RBC, WBC, MCH and MCHC (10.83 ± 0.16, 33.54 ± 0.54, 3.87 ± 0.06, 4.49 ± 0.18, and 32.20 ± 0.03) compared to those exposed for < 2 years (11.13 ± 0.05, 35.20 ± 0.27, 4.09 ± 0.06, and 4.40 ± 0.13) and unexposed Controls (13.25 ± 0.01, 43.14 ± 0.32, 4.61 ± 0.01, 5.62 ± 0.01, and 30.71 ± 0.01), respectively. The prevalence of anemia was higher among Subjects exposed to 2 or more years compared to Subjects exposed to < 2 years and the Controls. A significant positive correlation existed between the length of exposure and anemia and leucopenia.
Opute et al. (159)	Benin city, Edo State, Southsouth	Subjects: 20 PSAs; 5 Controls (students at the University of Benin) aged 18–25 years. 5 PSs.	Significant reductions in Hb, PCV, RBC counts, and WBC counts in the Subjects compared to the Controls. Urinalysis revealed high levels of bilirubin, urobilinogen, and nitrites in Subjects
Kalio et al. (160)	Port Harcourt, Rivers State, Southsouth	Subjects: 22 FPAs; Controls 22.	The mean ± standard deviation for Total protein was 60.19 ± 8. /l, Albumin 35.30 ± 3.30 g/l and Globulin was 24.89 ± 4.86 g/l in FPAs exposed to petroleum pollutants, while in the Controls, the values were Total protein 73.32 ± 1.41 g/l, Albumin 44.17 ± 0.23 g/l, and Globulin 29.15 ± 1.18 g/l, respectively. All parameters were significantly lower in FPAs than in Controls.
Adamu et al. (161)	Gombe, Gombe State, Northeast	Subjects: 90 apparently health roadside petrol dispensers and 90 matched Controls in Dukku and Bauchi Motor Parks in Gombe	The plasma level of uric acid (5.35 ± 0.9 mg/dl) in Subjects was significantly (*p* < 0.05) higher than the Controls (4.48 ± 0.9 mg/dl). No significant difference in the plasma levels of urea and creatinine.
Christian et al. (85)	Port-Harcourt, Rivers State, Southsouth	Subjects: 29 fuel filling attendants (20 males, 9 females); Controls 29 (20 males, 9 females) students of the University. Both Subjects and Controls were aged 19–52 years. 8 PSs.	The WBC, granulocytes, lymphocytes, and monocytes were significantly higher in subjects than in the Controls. Although platelets, serum lead, hemoglobin and PCV were also higher among the Subjects than the Controls, the difference was not statistically significant.
Ibeh et al. (162)	Nnewi, Anambra State, Southeast	Subjects: 50, including 25 PSAs (20 males and 5 females) and 25 (all males) AMs; Controls 50 occupationally unexposed persons recruited from amongst students. The number of PS not stated.	BLL and WBC were significantly higher in AMs than PSAs and controls. At the same time, Hb concentration, HCT, MCHC, MCV, MCH, and PLT count were significantly higher in Controls compared to PSAs. The MCV and MCH were negatively correlated with BLL in PSAs.
Ifeyinwa et al. (163)	Owerri, Imo State, Southeast	Subjects: 108 fuel pump attendants; Controls 108 shop attendants.	There was a statistically significant decrease in PEFR of fuel pump attendants (308.89 ± 51.34) compared with the shop attendants (350.46 ± 46.41).
Umegbolu et al. (164)	Awka, Anambra State, Southeast	Subjects: 35 (14 males and 21 females) petrol station pump attendants in the age ranges of 20–49 years; Controls 35 (14 males and 21 females) in the age range of 20–49 years. Both subjects and controls not exposed to cigarette and heavy alcohol consumption. 9 PSs.	There was a significant difference in micronuclei detection between the exposed and control groups. Beyond 2 years of exposure, the length of exposure had a weak positive correlation with the number of detected MN.
Ciroma et al. (165)	Kaduna, Kaduna State, Northwest	Subjects: 21 subjects (15 males and 6 females); Controls 20 (20 males and 5 females) non-occupationally exposed individuals. The number of PS was not stated.	A significantly lower FVC was observed in male subjects compared to male Controls. No significant difference between the exposed females and female Controls in terms of FEV1, FEV1% and PEFR
Kalio and Hanson (166)	Port Harcourt, Rivers State, Southsouth	Subjects: 32 PPAs; Controls 32 healthy non-petrol exposed office workers. The number of PS was not stated.	The mean anion gap in PPAs (22.85 ± 6.69 mmol/l) was significantly higher in Subjects than in the Controls (15.94 ± 3.53). Exposure to petroleum pollutants harms the production and excretion of protons and anions
Iyanda and Anetor (167)	Ibadan, Oyo State, Southwest	Subjects: 50 comprised 10 Group A male adult FFSA (who used the protective measures consistently while dispensing petroleum products) and 40 Group B FFSAs (who did not use protective gears); Control 30 Group C male adults not occupationally exposed to petroleum products. The number of PS was not stated.	In both Group A and Group B, activities or levels of ALP, AST, ALT, creatinine, urea, and bilirubin were significantly higher than the Controls (Group C). Contrariwise, total protein and albumin were significantly lower in Group A and Group B compared to Controls (Group C).
Obi et al. (88)	Onitsha, Anambra State, Southeast	Subjects: 80 gasoline station workers; Controls 80 healthy individuals. Number of PS not stated.	The mean methaemoglobin, BLL, and gamma-glutamyl carboxylase levels were significantly higher among the station workers than the Controls.
Anakwue et al. (168)^a^	Enugu, Enugu State, Southeast	Subjects: 164 PSAs, 175 AMs, 76 petrol tanker drivers aged 20–60 with a mean age of 38 ± 12 years; Controls 415 healthy non-occupationally exposed shop attendants with a mean age of 37 ± 11 years. The number of PS was not stated.	Statistically significant liver parenchymal echogenicity and liver size increases were seen in Subjects compared to Controls. These increased as the exposure duration increased. Out of 70 exposed workers with abnormal liver echo patterns, only 2.65% (*N* = 11) had alanine aminotransferase above the reference range.
Christian and Eze (169)	Port Harcourt, River State, Southsouth	Subjects: 29 PSAs; Controls 20 non-exposed healthy aged-matched individuals. 4 PSs.	The mean metHb level was significantly higher (5.676 ± 3.044) among the PSAs compared to the Controls (2.085 ± 0.815). Increased duration of occupational exposure to petroleum products was associated with an increase in metHb level
Iyanda (170)	Ibadan, Oyo State, Southwest	Subjects: 86 PPAs, including 30 males currently employed as PSAs with < 6 months of exposure work history (Group I), 34 male teenagers with work history not < 20 months and 22 male teenagers (Group III) who were former PSAs; Controls were 35 age-matched participants with no history of occupational exposure to fuel, or heavy metals. The number of PS was not stated.	The serum levels of Pb, As, nickel, cadmium, and aluminum of PPAs in Groups I and II were significantly higher compared with the Controls. The levels of lead and cadmium were significantly higher in Group III compared with controls. Headache and fatigue were the significant symptoms reported by Subjects in Groups I and II. Teenage PPAs were at risk of heavy metal toxicity despite quitting work at the petrol stations.
Dissi (171)	Kano, Kano State, Northwest	Subjects: 56 petrol pump attendants (PPAs); Controls 59 age, sex, weight, and height-matched employees from the bank. All participants were from 18 to 40 years of age. The number of PS was not stated.	Whereas the cardiovascular parameters did not differ between the PPAs and the Controls; significantly lower values of FVC (5.66L vs. 3.77L), FEV1 (5.10L vs. 2.93L), PEF (6.43L vs. 4.10L), Tiffeneau-Pinelli index (90.6 vs. 78.6%) and significant hypoxaemia (97.12 vs. 95.36%) were observed among the Subjects vs. the Controls.
Airhomwanbor et al. (172)	Ekpoma, Edo State, Southsouth	Subjects: 60 PSAs; Controls 40 healthy individuals. The number of PS was not stated.	There was a significant increase in the lead level but no significant difference in the levels of iron, zinc and copper of PSAs when compared with Controls.
Obeagu et al. (173)	Umuahia, Abia State, Southeast	Subjects: 50 PSAs and Controls 50 non-occupational exposed individuals. The number of PS was not stated.	The mean ± standard deviation of serum copper in PSAs was 0.796 ± 0.158 μ g/l and 1.216 ± 0.366 μg/l in the Controls. The mean ± standard deviation of serum selenium was 0.186 ±0.022 μg/l in PSAs and 1.029 ± 0.167 μg/l in the Controls. Interestingly, copper, selenium and zinc levels in PSAs decrease compared to copper, selenium, and zinc levels in Controls.
Ben-Chioma and Nwachuku (174)^a^	Obio-Akpor and Phalga, Rivers State, Southsouth	Subjects: 25 PSAs and 25 welders; Controls 20 healthy. Participants aged 15–40 years. The number of PS was not stated.	Serum levels of lead were higher in PSAs compared to the Controls. Cadmium levels were only higher among PSAs compared to Controls.
Adamu et al. (175)	Gombe, Gombe State, Northeast	Subjects: 90 apparently health roadside petrol dispensers and 90 age- and sex matched Controls	Higher level of oxidative stress in roadside petrol dispensers evidenced by significantly lower levels of mean plasma total antioxidant status (TAS) in Subjects (0.60 ± 0.33 mmol/L) compared to Controls (1.29 ± 0.25 mmol/L).
Ovuakporaye (176)	Ughelli, Delta State, Southsouth	Subjects: 150 PSAs; Controls 150 age and sex-matched individuals. Number of PS not stated.	The mean arterial pressure, regardless of the duration of years of dispensing petrol, was significantly higher among PPAs than the Controls. Compared to the Controls, the systolic and diastolic pressure were significantly higher among PPAs that have worked for more than 5 years.
Eze et al. (87)	Enugu, Enugu State, Southeast	Subjects: 60 PSAs; Controls 30 non-occupationally exposed shopkeepers. The number of PS was not stated.	The mean total WBC counts were different among PSAs exposed >2 years (4.74 ± 0.36 × 10^9^/L), PSAs exposed for < 2 years (5.36 ± 0.70 × 10^9^/l and the Controls (5.77 ± 0.70 × 10^9^/l) (*p* < 0.0001). The mean granulocyte count levels were statistically lower among PSAs exposed for >2 years (43.86 ± 10.34%) compared to controls (50.89 ± 7.62%). The mean level of copper was lower and mean level of lead were higher among the subjects than the Controls.
Anakwue et al. (177)^a^	Enugu, Enugu State, Southeast	Subjects: 415, including 164 PSAs, 175 Ams and 76 petrol tanker drivers aged 20–65 years, who have been exposed to petrol fumes for 5 years and more.; Controls were 415 age-matched shop attendants not occupationally exposed to petroleum distillates or solvents and who has been on the jobs for at least 5 years. The number of PS was not stated.	Increased echogenicity of the kidneys was observed in 21 Subjects which differed significantly from the findings in the Control group. In addition, the mean urea and creatinine levels were significantly higher in Subjects compared to Controls, although they did not exceed the general reference levels.
Okemuo et al. (178)	Enugu, Enugu State, Southeast	Subjects: 64 PSAs: controls 64 non-petrol attendants. The number of PS was not stated.	There was a significant reduction in FVC, FEV_1_, and PEFR in the petrol attendants compared to the control. There was also a significant increase in SBP, DBP, and respiratory symptoms in the petrol attendants compared to their control counterparts.
Anyiam et al. (179)	Onitsha, Anambra State, Southeast	Subjects: 80 gasoline station workers; Controls 80 students	RBC count, MCH, MCHC, PCV and platelet were significantly reduced in the Subjects compared to the Controls
Emokpae and Oyakhire (86)	Benin city, Edo State, Southsouth	Subjects: 60 PSAs (45 males and 15 females); Controls 30 healthy non-occupationally exposed individuals (21 males, 9 females).	Serum levels of FSH, LH, oestradiol, progesterone, and testosterone were significantly lower among PSAs than controls. The mean blood cadmium and lead levels were significantly higher among the PSAs compared to Controls.
Okeke and Kelechi (180)^a^	Nnewi, Anambra State, Southeast	The subjects were 30 cigarette smokers and 30 PSAs. Controls were apparently healthy individuals who neither smoke nor worked at PS. PSs Not stated	The mean COHb levels of smokers (0.83 ± 0.15) and PSAs (0.49 ± 0.08) were significantly higher when compared with the mean in the Controls (0.41 ± 0.01).
Ufelle et al. (181)	Enugu, Enugu State, Southeast	Subjects: 150 PSAs (53 males and 47 females); Controls 100 (50 males and 50 females) from the general population not occupationally exposed to petrol vapors. Both subjects and controls do not smoke a cigarette and do not have a cancerous illness. PSs Not stated	Compared to the Controls, the male PSAs had significantly higher levels of erythropoietin, interleukin-3, ALT, ALP, MCHC, and MCV and a significantly lower level of MCH. Female PSAs had significantly higher levels of interleukin-3, ALT, AST, ALP, MCHC, and MCV and significantly lower levels of MCH, platelets, Hb and HCT compared to Controls.
Onitsha et al. (182)	Yenagoa, Bayelsa State, Southsouth	Subjects: 30 PSAs; Controls 20 unexposed individuals. Both subjects and controls comprised 30 males and 20 females between the ages of 18–35 years. PSs 4	The mean serum calcium (2.40 ± 0.42) was lower in subjects than in Controls (2.62 ± 0.19). Whereas, the mean serum magnesium (0.83 ± 0.08) and the mean phosphorus (1.13 ± 0.17) were lower than the respective (0.86 ± 0.09 and 1.14 ± 0.22 in the Controls, these levels were not statistically significant.
Obeagu et al. (183)	Ondo, Ondo State, Southwest	Subjects: 50 PSAs.	No significant difference in PCV, WBC, Lym, Gran, RBC, Hb, MCV, MCH, MCHC, and PLT but there was significant difference in PDW (21.66 ± 30.88Fl vs. 52.02 ± 42.58fL) between male and female subject, respectively.
Anetor et al. (3)	Oye-Ekiti, Ekiti State, Southwest	Subjects: 50 gasoline dispensers (GDs); Controls 50 non-occupationally exposed participants from Oye local government area and environment. The number of PS was not stated.	Phenol was significantly higher in GDs compared to Controls. Cu, Fe, and Zn were significantly decreased in GDs compared to Controls. Phenol and Fe were negatively correlated. Heme and Zn were also negatively correlated to phenol.
Okeke et al. (184)	Nnewi, Anambra State, Southest	Subjects: 100 PSAs aged 18–30 years; Controls 50 healthy undergraduate students of the College of Health Sciences, Nnamdi Azikiwe University, Nnewi campus, aged 18–28 years. Number of PS not stated	The Prothrombin time was significantly shorter in the PSAs than in the control group. In comparison, the activated partial thromboplastin time was significantly longer in the PSAs than in the control group. The monocyte count was also significantly higher in PSAs than in the Controls. Also, among the PSAs, the males had significantly longer PT and APTT values compared to their female counterparts.
Eworo et al. (185)	Calabar, Cross-River State, Southsouth	Subjects: 50 gasoline station attendants; Controls 50 non-gasoline station attendants. Both Subjects and Controls were 18–60 years of age. The number of PS was not stated.	The body mass index, TPP, OSI and 8-OHdG levels were significantly higher, and TAC and PEFR were lower in Subjects than in the Controls. Positive correlations were observed between TPP and years at work, between TPP and OSI, and a negative correlation between TAC and OSI only in the Subjects.

^a^When studies of OHS involved PSWs and other artisans (auto-mechanics, panel beaters, automobile sprayers, list not exhausted) whose occupations also involve contact with petrol/petroleum products, the focus was primarily on the PSWs.

FSA, filling station attendant; PSA, petrol station attendants; PSWs, petrol station workers; PS, petrol station; DPR, department of petroleum resources; PPEs, personal protective equipment; PPAs, petrol pump attendants; FPAs, filling pump attendants; FFSAs, fuel filling station attendants; GDs, gasoline dispensers; AM, auto-mechanics; LBP, low back pain; LDL, low density lipoprotein; HDL, high density lipoprotein; VOCs, volatile organic compounds; OR, odds ratio; AOR, adjusted odds ratio; CI, confidence interval. FEV1, forced expiratory volume in the first second; PEF, peak expiratory flow-PEF; PEFR, peak expiratory flow rate; FVC, forced vital capacity; SBP, systolic blood pressure; DBP, diastolic blood pressure; Pb, lead; BLL, blood lead level; MetHb, methaemoglobin; TP, total protein; IgA, immunoglobulin A; IgG, immunoglobulin G; IgM, immunoglobulin M; ALT, alanine transaminase; AST, aspartate transaminase; ALP, alkaline phosphatase; MCH, mean cell hemoglobin; MCHC, mean cell hemoglobin concentration; MCV, mean cell volume;PCV, packed cell volume; Heme, hemoglobin; Hb, hemoglobin; HCT, haematocrit; WBC, white blood cell; RBC, red blood cell; PLT, platelet; Gran, granulocyte; Lym, lymphocyte; PDW, platelet distribution weight; SGPT, serum glutamic pyruvic transferases; SGOT, serum glutamic oxaloacetic transferases; ALAD, aminolevulinic acid dehydratase; EPP, erythrocyte protoporphyrin; AcChE, acetylcholinesterase; MN, micronuclei; As, arsenic; Cu, copper; Fe, iron; Zn, zinc; Ca, calcium; Mg, magnesium; Na, sodium; HCO_3_, bicarbonate; TAC, total antioxidant capacity; TPP, total plasma peroxides; OSI, oxidative stress index; 8-0HdG, 8-hydroxy-2-deoxyguanosine; COHb, carboxyhaemoglobin.

For most studies, the hematological indices were found to be lower among the PSWs compared to the controls that were not occupationally exposed to petrol liquid or vapors/fumes (142, 146, 153, 154, 158, 159, 162, 179). Contrariwise, Christian et al. (85) in Port-Harcourt, showed that white blood cells, granulocytes, lymphocytes, and monocytes were all elevated among PSWs compared to the controls. However, in Ibadan, Akintomiwa et al. (140) reported no significant difference in the blood levels of red blood cells, white blood cell, Hb and PCV between PSWs and the controls.

PSWs tended to have reduced pulmonary functions compared to the controls that were not occupationally exposed to petrol fumes/petroleum products (151, 155, 163, 171, 178). Anakwue et al. (168) described increased kidney echotexture among 21 of 36 PSWs in Enugu.

The four studies (136, 139, 143, 145) in Abeokuta, Southwest, described the effects of interventional 2 weeks ascorbic acid (500 mg daily) supplementation on some toxicities of chronic lead exposure in some occupationally exposed subjects including petrol station attendants. These studies reported that ascorbic acid supplementation can ameliorate chronic lead poisoning among occupationally exposed PSWs.

Umegbolu et al. (164) suggested the possibility of cancer risk in Awka, when the authors detected many micronuclei among 35 PSWs compared to controls not exposed to petrol. This risk was also found to increase beyond 2 years of exposure.

### 3.3 Air quality studies

Table 4 provides a summary of studies on air quality measurement. All the authors reported poor ambient air quality at the petrol stations. In addition, Olabisi (189) reported no known occupational health issues among the PSWs, although awareness of safety measures was poor at 10.0%. Oni and Ana (190) also reported a significantly lower mean peak expiratory flow rate (PEFR) among 100 PSWs in 20 petrol stations.

**Table 4:** Summary of air quality studies.

**References**	**Study site**	**Subjects and controls, and number of petrol stations sampled**	**Major findings**
Ekpenyong et al. (68)	Uyo, Akwa-Ibom State, Southsouth	Subjects: 117 PPAs; Controls were 118 age-matched women. Both subjects and controls were between 18 and 40 years.	The prevalence of menstrual disorders was 37.2 and 28.5% for Subjects and Controls, respectively. The menstrual cycle length and flow quantity were also significantly affected by petrol vapor exposure, with a 3.25 odd ratio for abnormal cycle length and 4.16 times odd for abnormal flow quantity. Menstrual disorders were also significantly more among the Subjects with more than 1 year of exposure to petrol fumes. There were also persistent low serum oestradiol levels and fluctuating levels of other reproductive hormones.
Okonkwo et al. (186)	Umuahia, Abia State, Southeast	Measured ambient air quality at the fronts, dispenser area and generator rooms of 3 filling stations	The average levels of volatile organic compounds (99.49 mg/m^3)^, carbon monoxide (5.48 mg/m^3)^, nitrogen dioxide (0.32 mg/m^3^) exceeded the FEPA air quality guidelines. The particulate matters (PM1 and PM2.5) were found to be at concentrations within FEPA air guideline.
Adebiyi et al. (187)	Ile-Ife, Osun State, Southwest	Measured ambient air PM2.5 and PM 10, and their elemental concentrations of Na, Mg, Al, Si, P, S, Cl, K, Ca, Ti, V, Cr, Mn, Fe, Ni, Cu, Zn, Br, Rb, Sr, Zr, Cs, Ta, W, and Pb in each PM at 4 PSs	The PM10 average values were higher than the 24 h WHO threshold limit of 50 mg/m^3^ in all 4 PSs, and the PM2.5 were higher than the WHO limits of 25 mg/m^3^ in all but one PS. All 25 elements were anthropogenic in both PM fractions, with concentrations exceeding the WHO standards
Lawal et al. (188)	Kaduna, Kaduna State, Northwest	It measured BTEX and other VOCs collected on an improvised sampler containing commercially activated charcoal in one petrol station	Presence of BTEX and other VOCs in the carbon disulphide extracts of the improvised sampler.
Olabisi (189)	Ilorin, Kwara State, Northcentral	Subjects: 90 PSAs in 30 petrol stations	The concentration of gases was LEL % (4.7187), hydrogen sulfide (0.7187%), carbon monoxide (8.4375%), oxygen (20.6875%), particulate matter-PM2.5 (35.1563 μg/m^3^), particulate matter- PM10 (37.3750 μg/m^3^), formaldehyde (0.3759 mg/m^3^), and volatile organic compounds (0.9531 mg/m^3^). While no health hazard was reported, 9 (10.0%) PSAs were aware of safety measures available at petrol stations. More awareness education on OSH is needed, and the provision of PPE for PSAs cannot be over-emphasized
Oni and Ana (190)	Ibadan, Oyo State, Southwest	Subjects: 100 FSAs in 20 petrol stations	The total mean PM 10 concentrations (μg/m3) in the morning was (43.7 ± 16.5) and (27.8 ± 7.9) in the afternoon, which were lower than the WHO guideline limit of (50 μg/m3). The total mean TVOC (ppm) in the morning (12.0 ± 3.4) and afternoon (5.6 ± 2.4) were also higher than the Occupational Safety and Health Administration (OSHA) guideline limit (3 ppm). The mean FEV1 of 1.63 ± 0.39 and the mean PEFR of 171.7 ± 45.9 were low among the filling station attendants.
Ana et al. (191)	Ibadan, Oyo State, Southwest	Subjects; 100 FSAs in 20 filling stations	Mean CO concentrations in the morning (15.4 ± 2.1 ppm) and afternoon (11.6 ± 1.4 ppm) were statistically higher than the WHO guideline of 9.0 ppm. The mean %COHb for FSAs (11.1 ± 2.6) was significantly higher than the WHO guideline of 2.5%. Symptoms observed included vomiting (13.4%) and nausea (14.9%).
Odilichukwu et al. (192)	Effurun, Delta State, Southsouth	10 cooking-gas/PSs	The petrol stations were within OSHA standard rating for O_2_ = 206,000-ppm as OSHA = 195,000-ppm, for CO_2_ = 23-ppm as OSHA = 5,000-ppm, for CO = 14-ppm as OSHA = 50, for NO = 15-ppm as OSHA = 25-ppm and SO_2_ = 0-ppm as OSHA = 5-ppm, whereas NO_2_ calculated was 18-ppm was higher than OSHA recommended value (5-ppm).

TVOC, total volatile organic compounds; LEL, lower explosive limit; PM, particulate matter; FSAs, filling station attendants; PSAs, petrol station attendants; WHO, world health organization; COHb, carboxyhaemoglobin; PEFR, peak expiratory flow rate; FEV1, forced expiratory volume in 1 second; OSHA, occupational safety and health; O_2_, oxygen; CO, carbon monoxide; CO_2_, carbon dioxide; NO, nitrogen monoxide; NO_2_, nitrogen dioxide; SO_2_, Sulfur dioxide; VOCs, volatile organic compounds; of Na, sodium; Mg, magnesium; Al, aluminum; Si, silicon; P, phosphorus; S, Sulfur; Cl, chloride; K, potassium; Ca, calcium; Ti, titanium; V, vanadium; Cr, chromium; Mn, manganese; Fe, iron; Ni, nickel; Cu, copper; Zn, zinc; Br, bromine; Rb, rubidium; Sr, strontium; Zr, zirconium; Cs, cesium; Ta, tantalum; W, tungsten; and Pb, lead; PM, particulate matter; FEPA, Food and Environmental Protection Act 1996.

Only Ekpenyong et al. (68) in Uyo, measured and quantified BTEX in the breathing zones of female PSWs. Ekpenyong et al. (68) reported a higher mean concentration of BTEX compounds in female petrol attendants than in ambient air sampled a few kilometers from the gasoline stations. Ekpenyong et al. (68) also found that petrol vapors exposure significantly affected the menstrual cycle length and flow quantity. There were also persistent low serum oestradiol levels, and the mean benzene concentration among the PSWs was more than the threshold limit value for benzene (0.5 ppm). Okonkwo et al. (186) in Umuahia found that levels of volatile organic compounds, methane, carbon monoxide, nitric oxide exceeded the FEPA air quality guidelines. However, the particulate matters (PM_1_ and PM_2.5_) were found to be at concentrations within FEPA air guideline (193).

Lawal et al.'s (188) work in Kaduna, was a preliminary study to test the effectiveness of an improvised air sampler to capture VOCs and BTEX in ambient air around the petrol station. The researcher used commercially available activated charcoal as an adsorbent media, and they demonstrated the presence of BTEX in the ambient air of a petrol station, with the prospect of planning a more detailed study in the future.

## 4 Discussion

### 4.1 Occupational health and safety at Nigeria's petrol stations

The observational studies highlight that the knowledge, attitude, and practices (KAPs) of PSWs on workplace hazards, health problems, workplace accidents, safety measures, and PPE vary from one study setting to another. The observational and analytical studies suggest that exposure to petrol fumes/petroleum products of the PSWs results in adverse effects on the respiratory function indices and the hematologic, immunologic, hepatic, and renal systems of the PSWs. The air quality studies confirm the poor air quality of the NPSs, including the adverse effects of BTEX exposure on the reproductive system of female PSWs. Although the KAPs of OHS in NPSs vary from one study setting to another, generally, the consensus is that safety practices are poor and that awareness about hazards and PPE and using PPE is sub-optimal. The Nigerian PSWs are at an increased risk of exposure to BTEX, petrol fumes, and petrol liquid.

Possible reasons for poor safety practices and sub-optimal awareness about hazards and PPE, and the usage of PPE among PSWs in Nigeria include lack of adequate information about hazards at petrol stations and lack of knowledge about the capacities of PPE to reduce exposure to hazardous BTEX/petrol fumes/liquid petrol (69). Other reasons are inadequate training on safe practices before employment and ineffective law enforcement, which promotes the lackadaisical attitudes of employers toward OHS issues (131).

Although Ekpenyong et al. (68) document the toxicity of BTEX on menstruation and reproductive hormonal profiles, the researchers did not measure BTEX biomarkers that would have confirmed exposure to BTEX through multiple exposure pathways. Thus, their work suffered some drawbacks as workers' exposure to BTEX through the skin or gastrointestinal tract was not accounted for.

In Nigeria, because many filling stations fail to adhere to the stipulations of Nigeria's Department of Petroleum Resources (DPR), the regulatory body, an increasing number of PSWs are exposed to hazards at filling stations (129, 133). During the refueling of automobiles, the atmospheric concentration of gasoline vapor is between 20 and 200 ppm (194, 195). This amount is higher when a long queue of automobiles needs refueling (195), a common occurrence in Nigeria because of perennial fuel scarcity (4). Furthermore, exposure of the Nigerian PSWs to BTEX occurs from exhaust fumes from generator sets used in powering the petrol dispensing pumps. This is a common finding as the DPR requires that every filling station have one electric generator set for its normal operations (196). The need for a canopy over petrol pumps at the petrol station is another expectation of the DPR (196). Unfortunately, a canopy over the pumps increases the ambient flux of BTEX around the station (197). Moreover, the harsh economic situation in Nigeria forces most motorists to go about on near-empty petrol tanks with the attendant voluminous petrol fumes in the head-spaces when they come for refueling at the petrol stations (151, 171). In addition, Adeniyi (151) described a situation where petrol pump attendants are constrained to stay put by the vehicle as the vehicle tanks get filled, as most fuel pumps can only dispense petrol with the attendants holding the nozzles.

The effect of temperature on the vaporization of BTEX has been conflicting (52, 198, 199). While Periago et al. (199) and Kitwattanavong et al. (52) demonstrated that temperature was positively correlated to ambient BTEX concentrations in gasoline stations; Moolla et al. (198) reported a negative correlation between temperature and ambient BTEX concentrations in diesel station. Thus, although the concentration of BTEX is usually lower in warmer months (31, 41) and higher in colder winter months (31, 41), these findings may not be universal and many other variables have to be accounted for. Whereas, the cold temperature layer during winter hindered the dilution of BTEX and thus led to higher concentrations of BTEX in the atmosphere, while intense photochemical activity and dilution due to the increase in the mixing layer depth in summer led to lower concentrations of BTEX (41). Nigeria is in tropical Africa, with high temperatures for most of the year, hence, a strongly powered study that will test the effect of temperature and other meteorological conditions is warranted.

Lead (tetraethyl and tetramethyl lead) and BTEX are used as petrol antiknock and lubricating agents to improve machine efficiency (24). Although it was officially announced that lead had been phased out in Nigeria in 2004, the 2017 standard for Nigeria's gasoline still contains lead of 50 ppm and BTEX of 2% v/v (see text footnote 2). Most lead is emitted from motor vehicles as inorganic particles (200), and leaded gasoline causes more exposure to lead than any other known source (201). Furthermore, lead and BTEX in gasoline cause overlapping health hazards, including gastrointestinal and hematological disturbances, hepatic and renal damage, hypertension and neurological disorders (21, 200, 202). The acute and chronic health symptoms of lead and BTEX are indistinguishable from each other, and attribution would have to be done by specific measurements of lead and BTEX among occupationally exposed workers. Acute lead exposure may cause gastrointestinal disturbances (anorexia, nausea, vomiting, abdominal pain), hepatic and renal damage, hypertension and neurological effects (malaise, drowsiness, encephalopathy) that may lead to convulsions and death (203). Chronic lead exposure effects include hematological effects (anemia), neurological disturbances (headache, irritability, depression, lethargy, convulsions, muscle weakness, ataxia, tremors and impaired hearing), gastrointestinal disorders (abdominal colic), and kidney dysfunction (204). These symptoms overlap with those shown in Table 1, resulting from acute and chronic exposures to BTEX (43–46). Thus, the works of Alasia et al. (83), Bambgose et al. (84), Christian et al. (85), Emokpae and Oyakhire (86), Eze et al. (87), Obi et al. (88), and Onuegbu et al. (89) which assessed the association of blood levels of lead and some clinical and biochemical changes among workers occupationally exposed to lead should be interpreted cautiously, as confounding exposure to BTEX cannot be excluded. Nevertheless, while children are especially vulnerable to the neurotoxic effects of lead (204), BTEX are the most commonly exposed VOCs among workers at petrol stations (21, 205). In addition, compared to benzene, which the IARC has classified as human carcinogen Group 1 causing acute myeloid leukemia and acute non-lymphocytic leukemia (43), inorganic lead compounds are probably carcinogenic to humans (Group 2A) (206) and organic lead are not classifiable in human carcinogenicity (Group 3) (206). Thus, while efforts to completely phase out lead in gasoline in Nigeria continue, future research efforts on BTEX exposures at NPSs should be considered favorably, as benzene remains the most toxic chemical additive in gasoline (207, 208).

### 4.2 Challenges of safeguarding occupational health and safety at Nigeria's petrol stations

Safeguarding the OHS of workers at NPSs is dependent on two prerequisites. First, there will be regulations and policies to guide the OHS of workers at the petrol stations. Second, there would be efficient and effective enforcement modalities of regulations and policies on OHS. Regarding the OHS of workers in NPSs, the two prerequisites are sub-optimal (72, 209). The Nigerian constitution statutorily empowers the DPR to ensure compliance with the oil and gas industry's regulations, guidelines, and laws (196).

Furthermore, since August 2021, and backed by Nigeria's Petroleum Industry Act of 2021 (210), the Petroleum Products Pricing Regulatory Agency (PPPRA) and the Petroleum Equalization Fund Management Board (PEFMB) have been merged with the Midstream and the Downstream Divisions of the DPR to form the Nigerian Midstream and Downstream Petroleum Regulatory Authority (NMDPRA) (210). The former DPR now operates under the NMDPRA (210). The NMDPRA, known as “The Authority”, statutorily provides a legal, governance, regulatory and fiscal framework for the Nigerian Petroleum Industry (210). The NPSs are retail outlets for petroleum products and they operate under the Downstream Division of the NMDPRA. The objectives of the PIA are to provide for the safety standards to be observed during midstream and downstream petroleum operations; (b) regulate safety and occupational health in Nigerian midstream and downstream petroleum operations; (c) set out the permits, authorizations, and fees for such midstream and downstream petroleum operations; and (d) provide sanctions, penalties, and administrative fines for failure to comply with these Regulations (210).

Table 5 shows the DPR guidelines regarding the sitting and construction of petrol stations in Nigeria (196). The DPR introduced the Minimum Industry Safety Training for Downstream Operations (MISTDO) as part of the Safety Audit Clearance policy launched to drive safety in the downstream sector. MISTDO is the basic safety training that is compulsory for all workers in the downstream sector of the Nigerian oil and gas industry (211). Regardless of the extant legislation and regulations on OHS in Nigeria, the consensus by observers is that OHS still needs improvement (72). The enforcement of the DPR guidelines needs thorough institutional improvements (212, 213). Owners of petrol stations have refused to adhere to proper land uses characterized by careless constructions, abnormally chaotic locations of petrol stations in residential areas, and over-concentration of petrol stations in one part of the urban cities, infractions that pose significant hazards to the health of workers, motorists and the people residing in surrounding environments to the petrol stations (212, 213). The poor sitting conditions of Nigeria's petrol stations also cause traffic congestion, air pollution, and fire hazards (72, 214). The abysmal state of OHS in Nigeria is attributable to the poor enforcement of OHS regulations and the need for more content and scope of the extant OHS regulations (215, 216).

**Table 5:** The Department of Petroleum Resources guidelines on construction of petrol stations in Nigeria (196).

A petrol station should have: • At least 3 underground storage tanks and 3 dispensing pumps (one each for petrol, kerosene, and diesel), • An office building with 2 office rooms, sales room, toilet, and lube bay/store (Optional) • A well-concreted forecourt • An “IN/OUT” driveway • A wall fence demarcating the station (minimum height of 1.5 m high) • A good drainage network that will not discharge into rivers or streams. • A standard canopy over petrol pumps (mandatory) • An accessible potable water source, and available safety facilities at the petrol stations • These safety measures should include fire extinguishers (with a current fire certificate and evidence of trained attendants on fire safety), sand buckets, a strategic display of “NO SMOKING” warning signs, an underground tanks pressure test report, and a certificate of leak detection test. • A petrol station should not lie within a pipeline or national electricity grid tension cable Right of Way (ROW) • The distance from the edge of the road to the nearest pump will not be < 15 meters. • The total number of petrol stations within 2 kilometers stretch of a petrol station on both sides of the road will not be more than four. • The distance between two petrol stations will not be < 400 meters

In general, the gravity of penalties stipulated by OHS laws in Nigeria is insignificant and cannot deter offenders from fouling the regulations (216, 217). For example, the penalty stipulated by the Workman's Compensation Act is as low as 2000 Naira (2.53 United States Dollars, at 2023 rates of 0.0013 USD to 1 Naira) or the premium payable for 1 year (whichever is greater) when an employer fails to insure the employees against death or injuries (217). Regarding the Midstream and Downstream Petroleum sectors, the penalty is stiffer. A licensee who fails to comply with any of the provisions of the safety regulations shall, in addition to the sanctions, fines and penalties contained in the Act, be liable to an administrative penalty of not more than USD 250,000 (210). Any permit or authorization granted to that licensee or holder may be suspended or revoked (210). In addition, a manager who fails to comply or ensure compliance with these Regulations is liable to an administrative penalty issued by the Authority of not more than N5,000,000, an equivalent of 6,314 USD (210).

Furthermore, Nigeria's slow and ineffective judicial process discourages workers from seeking redress in incidents that infract OHS (217). Moreover, because the labor supply often overshadows demands, employers tend to become overlords with a penchant for disregarding the extant regulation on OHS (217). This power imbalance also makes it difficult for workers to demand their rights in the workplace (217). Another barrier to enforcement is ignorance about workers' rights and privileges. Both employees and employers need to become more familiar with the Factories Act and other extant laws that define OHS, rights, privileges, and expectations at the workplace (217).

Apart from the DPR, other pieces of Nigerian legislation safeguard Nigerian workers' safety, health, and welfare at petrol stations. These legislations include the Constitution of the Federal Republic of Nigeria 1999, the Labour Act of 2004, the Factories Act of 2004, the Employee Compensation Act of 2010, the Minerals Oil Safety Regulation of 1999, and the Harmful Waste Act of 1990 (134, 217). However, regardless of these extant Acts, Laws, and Regulations on OHS in Nigeria, the consensus by reviewers is that the OHS of Nigerian workers is still poor, and by extension, this includes Nigerians working at petrol stations (72, 134, 217).

### 4.3 Factors that determine the vulnerability of petrol station workers to petrol/BTEX exposure at petrol stations

**Figure 3:**
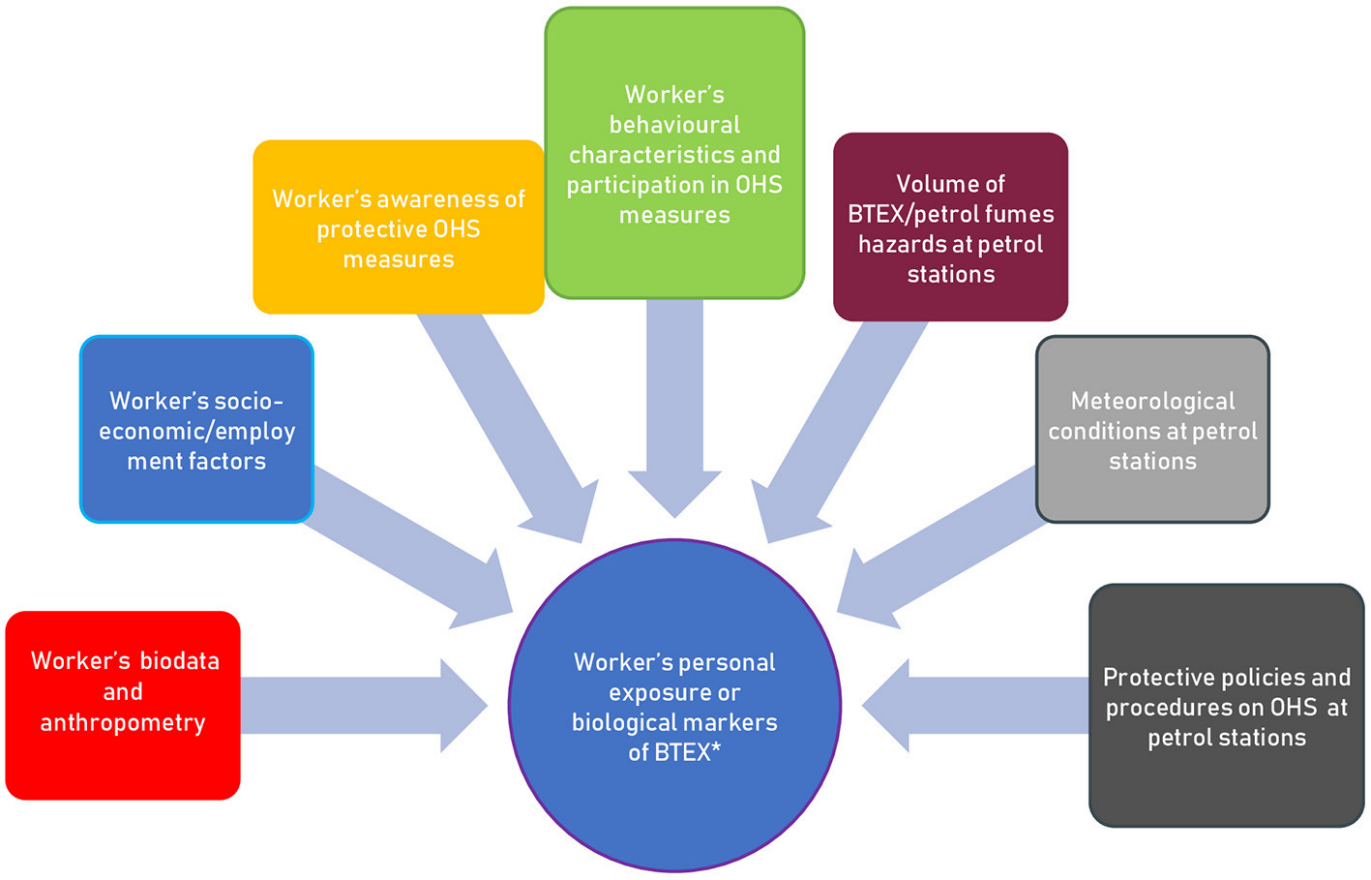
The seven risk factors that determine the vulnerability of petrol station workers to petrol/BTEX exposure at petrol stations. OHS, occupational health and safety. *Presence of confounding environmental source of BTEX: means of transport to work (walk, bicycle, motorcycle, commercial vehicle, personal vehicle), time spent in gelling to work, exposure to tobacco smoke, alcohol consumption, household of the PSWs including beating and cooking activities (gas, kerosene, firewood, animal dung), building finishing: inside painted or not, household storage of petrol, paint, pesticides, fertilizers and herbicides, household use of generator set, household use of varnishes, finger nail polish, glues at home, cleansing agents, and shoe polish, engagement in other jobs apart from working at petrol stations (painter, vulcanizer, mechanics, farmer).

In occupational epidemiology, the vulnerability of PSWs' exposure to petrol fumes or petrol liquid or BTEX can be contextualized as a dependent outcome or variable (personal monitoring or biological markers of BTEX) and the independent but interrelated risk factors. These independent factors determine PSWs' exposure to chemical hazards, including petrol/BTEX. The literature review of studies on occupational exposure to petrol or other petrochemicals containing BTEX by the authors identify seven risk factors of petrol station workers that determine their vulnerability to petrol/BTEX exposure at petrol stations as shown in Figure 3.

Table 6 depicts some studies that describe the risk factors that may determine the vulnerability of petrol station workers to petrol/BTEX exposure at petrol stations.

**Table 6:** Studies of risk factors that may determine the vulnerability of petrol station workers to petrol/BTEX exposure at the petrol stations.

**References**	**Risk factors**
Baghani et al. (218); Cezar-vaz et al. (55); Santiago et al. (63); Tunsaringkarn et al. (64); Ekpenyong and Asuquo (219); Xiong et al. (220); Tongsantia et al. (221)	Biodata/anthropometry
Al-Harbi et al. (20); Campo et al. (54); Cezar-vaz et al. (55); Chaiklieng et al. (65); Esmaelnejad et al. (222); Karakitsiosa et al. (223)	Socioeconomic/employment factors
Alves et al. (53); Tunsaringkarn et al. (64)	Awareness of protective OHS measures
Al-Harbi et al. (20); Alves et al. (53)	Behavioral characteristics and participation in OHS measures
Allahabady et al. (224); Al-Harbi et al. (20); Baghani et al. (218); Chaiklieng et al. (65); Chaiklieng (202); Esmaelnejad et al. (222); Keretetse et al. (225); Kitwattanavong et al. (52); Periago and Prado (226); Periago et al. (199)	Volume of BTEX/petrol fumes hazards at petrol stations
Abbasi et al. (42); Allahabady et al. (224); Baghani et al. (218); Cruz et al. (56); Jiang et al. (41); Karakitsioss et al. (223); Kitwattanavong et al. (52); 223; Yu et al. (31); Zheng et al. (49)	Meteorological conditions at petrol stations
Allahabady et al. (224); Huy and Oanh (227); Karakitsiosa et al. (223); Kuranchie et al. (21); Kitwattanavong et al. (52); Periago and Prado (226); Rahimpoor et al. (61)	Protective policies and procedures on OHS at petrol stations

OHS, occupational health and safety; PPE, personal protective equipment.

Efforts to reduce human exposure to BTEX have included reducing BTEX emissions at petrol stations in Europe by adopting vapor recovery systems (VRS) and by avoiding vapor losses during fuel transfer (228); restricting benzene composition in gasoline in Europe to 1% (v/v) (18) and to 0.62% (v/v) in the United States (19); and by standardizing benzene concentration in ambient outdoor air in Europe to 5 μg.m^−3^ (229). Table 7 shows the OELs of some professional institutions and countries (61). Unfortunately, there are no known national standards for OELs to BTEX in Nigeria (16). Instead, Nigeria's National Environmental Regulations on Air Quality Control (230) has some air quality standards for criteria for pollutants and air toxics, as shown in Table 8. Thus, PSWs in Nigeria are continuously being exposed to a work environment that exposes them to unmitigated levels of BTEX/petrol vapors.

**Table 7:** Occupational existing limits of benzene, toluene, ethylbenzene and xylene of some professional institutions and countries (61).

**Institution or country**	**Benzene mg/m^3^ (ppm)**	**Toluene mg/m^3^ (ppm)**	**Ethylbenzene mg/m^3^ (ppm)**	**Xylene mg/m^3^ (ppm)**
ACGIH, USA	1.6 (0.5)	75.37 (20)	86.84 (20)	434.19 (100)
OSHA, USA	3.19 (1)	376.85 (100)	434.22 (100)	434.19 (100)
NIOSH, USA	0.32 (0.1)	753.7 (0.1)	434.22 (100)	434.19 (100)
Australia	3.19 (1)	188.43 (50)	434.22 (100)	347.39 (80)
Brazil	–	293.94 (78)	338.69 (78)	338.67 (78)
Canada	1.6 (0.5)	75.37 (20)	86.84 (20)	434.19 (100)
Japan	3.19 (1)	188.43 (50)	86.84 (20)	217.1 (50)
South Korea	3.19 (1)	–	–	–
MAK, Germany	–	188.43 (50)	86.84 (20)	217.1 (50)
AGS, Germany	1.93 (0.6)	–	–	–
Netherlands	0.7 (0.22)	150.74 (40)	214.07 (49.3)	217.1 (50)
Polands	1.6 (0.5)	99.87 (26.5)	198.87 (45.8)	99.86 (23)
United Kingdom	3.19 (1)	188.43 (50)	434.22 (50)	217.1 (50)
European Union	0.32 (0.1)	188.43 (50)	434.22 (100)	217.1 (50)
Iran	1.6 (0.5)	75.37 (20)	86.84 (20)	434.19 (100)
Turkey	0.32 (0.1)	188.43 (50)	434.22 (100)	217.1 (50)

ACGIH, American Conference of Governmental Industrial Hygienists; AGS, German Committee on Hazardous Substances (Ausschuss für Gefahrstoffe); MAK, German maximum workplace concentrations; NIOSH, National Institute for Occupational Safety and Health; OSHA, Occupational Safety and Health Administration; ppm, part per million.

**Table 8:** Ambient air quality standards for criteria pollutants and air toxics in Nigeria (230).

**Ambient air concentration TWA**	**Sulfur dioxide (S0_2_) μg/m^3^**	**Nitrogen dioxide (NO_2_) μg/m^3^**	**Carbon monoxide (CO) μg/m^3^**	**Particulate Matter (PM10) μg/m^3^**	**Particulate Matter (2.5) μg/m^3^**	**Oxone (O_3_) μg/m^3^**	**Lead (Pb) μg/m^3^**	**Arsenic (As) μg/m^3^**	**Nickel (Ni) μg/m^3^**	**Cadmium (Cd) μg/m^3^**	**Ammonia (NH_3_) mg/m^3^**
Annual	80	80	–	60	20	–	1.0	6,000	20,000	5,000	0.2
24 h	120	120		150	40	–	1.4	–	–	–	0.6
8 h	–	–	5	–	–	100	–	–	–	–	–
1 h	350	200	10	–	–	180	–	–	–	–	–

TWA, time weighted average.

### 4.4 Protective strategies against exposure to BTEX/petroleum products at petrol stations

Providing protective and interventional strategies against BTEX exposure at the NPSs can adapt from the measures applied in other built environments (231). These strategies include administrative, environmental/engineering, and personal protective measures (231). Administrative controls are policies and procedures put in place and implemented by petrol station managers/DPR that will reduce the vulnerability of PSWs to BTEX exposure. Environmental controls at the petrol station focus on engineering strategies that collect and prevent BTEX and other vapors from escaping and polluting the atmosphere (vapor recovering), as well as those designs that destroy the collected BTEX and other vapors (vapor destroying). Vapor recovery occurs in two stages (227, 232). The first stage is when underground storage tanks are refilled by tanker trucks. The second stage is when motor vehicles' tanks are being refueled (227, 232). At both stages, gasoline vapors in the head-spaces of empty or partially empty storage tanks and vehicles' tanks rise and pollute the atmosphere as liquid petrol is transferred into the tanks (227, 232). USEPA Stage I recovers petrol vapors in the first stage using two hoses (227, 232). The first hose transfers gasoline from the tanker truck to the storage tank (227, 232). The second hose simultaneously collects gasoline vapors being displaced out of the storage tank and back to the empty tanker truck (227, 232). The displaced vapors can then be collected for incineration, converted to liquid, and returned to the storage tank (227, 232). USEPA Stage II is a balance recovery system that uses a vapor recovery nozzle with a guard at the root of the filling pipe and recovery holes on the filling nozzle pipe. The guard fits tightly to the vehicle's tank preventing the escape of displaced gasoline vapors from the head-space during refueling. The recovery holes return gasoline vapors into the underground tank. Stage II control cuts the escape of gasoline vapor emissions by up to 88–92% (227, 232).

**Figure 4:**
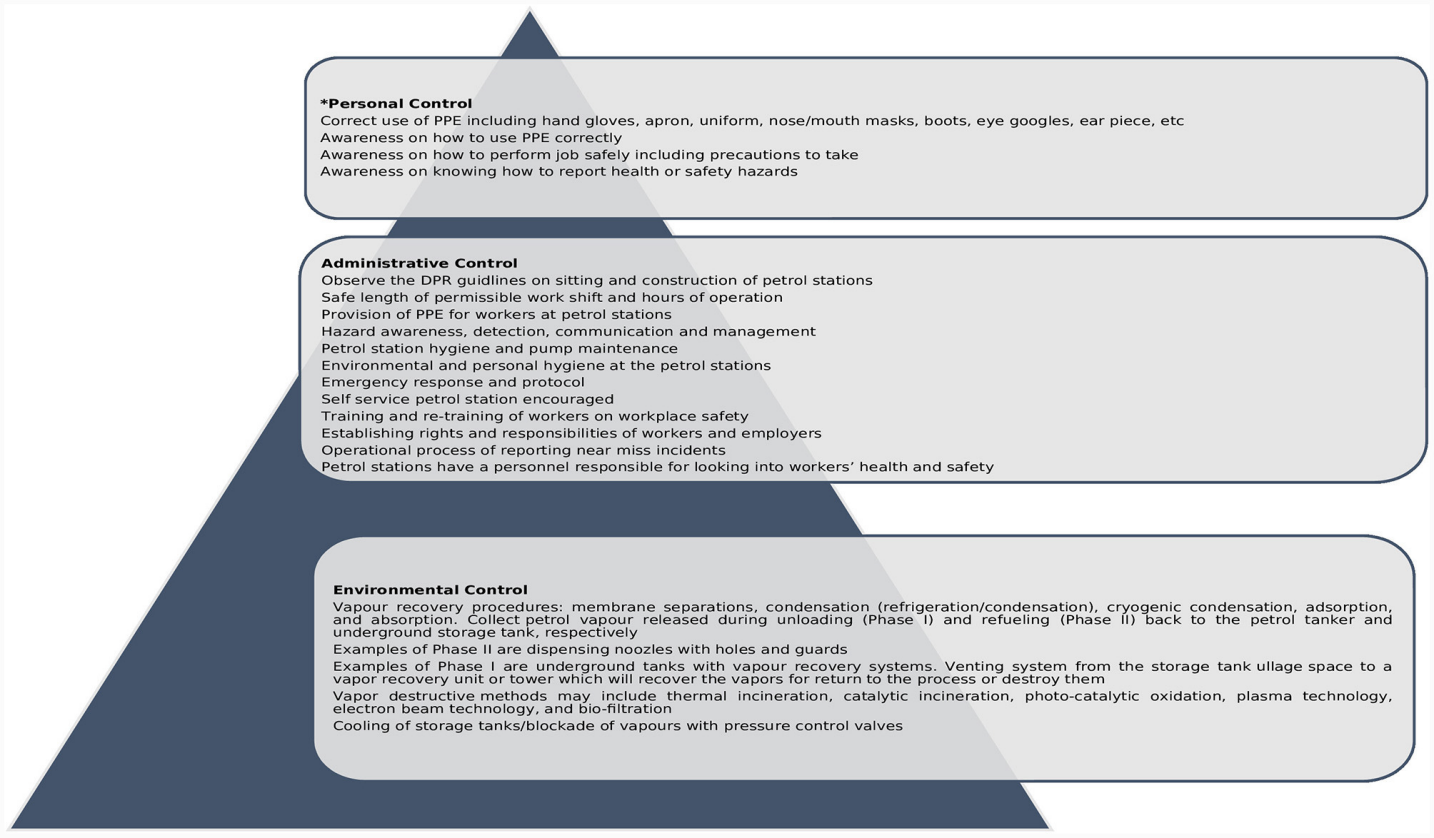
Hierarchy of control measures to protect workers at petrol stations. PPE, personal protective equipment; DPR, department of petroleum resources, *while personal control may be at the “tip of the iceberg”, the environmental control at the base is far more important.

Personal protection controls are used in high-risk environments and emergency scenarios as the last resort where administrative and environmental controls cannot adequately offer protection (231). Thus, while personal protection controls may be the most visible form of strategy (appearing at the 'tip of the iceberg), they are not the most important. Examples of personal protection include the use of PPE and personal hygiene. Figure 4 summarizes the strategies to control exposure to BTEX in Nigeria's petrol station-built work environment. Unfortunately, until a safer substance is available to serve as an alternative additive for petroleum products, physically removing (elimination) or replacing (substitution), BTEX cannot form an integral part of the hierarchy of controls of exposures to BTEX at this time.

### 4.5 Research gaps and future research directions

Despite the available research on the OHS of PSWs at NPS, further research on BTEX exposure and pollution in Nigeria must be emphasized. Table 9 depicts some areas of research that need to be developed to improve public health safety from BTEX pollution in Nigeria.

**Table 9:** Highlights of future research areas and research focus on BTEX pollution in Nigeria.

**Research area**	**Research focus**
Comprehensive research of BTEX exposure in petrol stations	• Measure and quantify BTEX exposure via passive and active sampling in ambient air of petrol stations and among PSWs. • Determine the health risk assessment of PSWs in Nigeria's petrol stations by estimating cancer and non-cancer risks to BTEX exposure. • Confirm the burden of exposure to BTEX among PSWs by measuring urinary metabolites of BTEX. • Determine the correlation of BTEX exposure to respiratory symptoms and other adverse health outcomes. • Determine the relationships between BTEX exposures and meteorological parameters at different seasons and regions of Nigeria. • Determine the potential sources of BTEX compounds at petrol stations by doing correlation and ratio analyses. • Determine the baseline BTEX concentrations in ambient air in a national study that may help to establish OELs for BTEX • Measure and compare BTEX exposures among comparators of non-occupationally exposed participants
Develop appropriate technologies that will reduce human exposure to BTEX at petrol stations	• Develop vapor abatement technologies and vapor abatement engineering that will limit the exposure of PSWs to BTEX • Vapor recovery procedures may include membrane separations, condensation (refrigeration/condensation), cryogenic condensation, adsorption, and absorption. • Vapor destructive methods may include thermal incineration, catalytic incineration, photo-catalytic oxidation, plasma technology, electron beam technology, and bio-filtration.
Study the exposures of other Nigerians who are occupationally exposed to BTEX pollution	• Measure and quantify in-vehicle BTEX in public transport vehicles and among public transport drivers. • Measure and quantify BTEX exposure via passive and active sampling among traffic policemen. • Determine the health risk assessment of public transport drivers and traffic policemen by estimating cancer and non-cancer risks to BTEX exposure. • Determine the correlation of BTEX exposure to respiratory symptoms and other adverse health outcomes.
Qualitative study (QS) or mixed-method study on perception and awareness of OHS at Nigeria's petrol stations	• Aim to gain an in-depth understanding of the OHS vulnerability at Nigeria's petrol stations. • The QS will seek to know perceptive, experiences, and attitudes regarding OHS vulnerability at the Nigeria's petrol stations. • The QS will include interviews of the regulators (Key Informant Interviews-KIIs) at the Department of Petroleum Resources (DPR) and focused group discussion (FGD) among petrol station workers (PSWs) and petrol stations' owners.
The role of artificial intelligence (AI) in determining the risk of BTEX air pollution	• Derive the mathematical modeling of airborne BTEX in private and public transportation including increased risk of exposure in traffic congestion and urbanization. • Determine the novel role of AI in tracking and dispersing of BTEX from various anthropogenic sources. • Determine the contribution of remote sensing and geospatial technologies as a tool for predicting vulnerabilities and tracking dispersion of BTEX pollutant. • Determine the geospatial dispersion and proximity of petrol stations to residential, educational institutes and commercial centers.
What is the contribution of BTEX pollutant from petrol stations on water pollution?	• Determine the BTEX contamination of surface (rivers or streams) and underground water around petrol stations. • Determine the source apportionment of BTEX, from petrol stations and non-petrol station sources. • Determine to what extent BTEX pollutant has altered the chemical, biological and physical nature of water. • Determine the contribution of BTEX pollution to water shortage and water recovery.
What is the contribution of BTEX pollutant from petrol stations on the surrounding top and underground soil?	• Determine the BTEX contamination of surface (topsoil) and underground soil around petrol stations. • Determine the source apportionment of BTEX contamination, from petrol stations and non-petrol station sources. • Determine to what extent BTEX pollutant has altered the natural structure, quality and composition of soil (BTEX, lead and other heavy metals). • Determine the contribution of BTEX pollution to agricultural soil wastage and soil recovery.
What is the feasibility of having self-service petrol stations in Nigeria?	Determine the financial implication of having self-service petrol stations in Nigeria to the petrol stations' owners.
Investigate biomarkers of susceptibility in subjects occupationally exposed to air pollution	Determine the role of genetic susceptibility and its relationship with exposure to BTEX xenobiotics.

PSWs, petrol station workers; OHS, occupational health and safety; DPR, department of petroleum resources; QS, qualitative study; FGD, focused group discussion; KII, key informant interview; AI, artificial intelligence; OELs, occupational exposure limits.

Controlling air pollution from BTEX and other hazardous air pollutants is an essential component of OHS, which forms an integral part of attaining the Sustainable Development Goals (SDG) 3 and 11 (233), of which the WHO is the custodian (234). The targets of the SDG relevant to ambient air pollution include SDG target 3.9.1 (it calls for a substantial reduction in deaths and illnesses from air pollution) and SDG target 11.6.2 (it aims to reduce the environmental impact of cities by improving air quality) (234).

## 5 Conclusion

The available literature revealed that OHS knowledge, attitudes, and practices in NPSs vary from one Nigeria's study setting to another. Generally, the consensus is that safety practices, awareness about hazards and PPE, and the usage of PPE among PSWs failed to live up to expectations. The Nigerian PSWs are at an increased risk of exposure to BTEX, petrol vapor, and petrol liquid. Currently, regulatory bodies' effectiveness and accountability in enforcing OHS regulations in NPSs leave much to be desired. Understanding the OHS of NPSs would inform future initiatives, policies, and regulations that would promote the health and safety of workers at NPSs. However, future studies need to be conducted that will describe and safeguard PSWs and other Nigerians who are occupationally exposed to BTEX pollution. Controlling air pollution from BTEX and other hazardous air pollutants is an essential component of OHS and integral to attaining the Sustainable Development Goals (SDG) 3, 7, and 11.

## 6 Limitations of study

In this study, it is essential to remember that while studies on OHS at NPS are reported based on the six geopolitical regions of Nigeria, we need to be cautious in interpreting and drawing conclusions from them. It is essential to note that while the results of these studies are factual for the specific settings where they were conducted, caution in generalizing the results to the entire geopolitical region of Nigeria needs to be emphasized.

## Author contributions

EA: Conceptualization, Data curation, Formal analysis, Methodology, Resources, Writing—original draft, Writing—review & editing. ZN: Conceptualization, Methodology, Resources, Supervision, Writing—review & editing. CW: Conceptualization, Funding acquisition, Project administration, Resources, Supervision, Writing—review & editing.

## Footnotes

^1^Nigerian Industrial Standard (NIS 948-2017)(2017). Standard for Diesel Fuel (AGO). file:///D:/NIS-948-2017.pdf.

^2^Nigerian Industrial Standard (NIS 116:2017). (2017). Standard for Premium Motor Spirit (Petrol). file:///D:/NIS-116-2017.pdf [accessed June 2, 2022].

^3^Nigeria-The World Factbook (2023). Available online at: https://www.cia.gov/the-world-factbook/countries/nigeria/ (accessed January 2, 2023).
